# Transcriptional firing helps to drive NETosis

**DOI:** 10.1038/srep41749

**Published:** 2017-02-08

**Authors:** Meraj A. Khan, Nades Palaniyar

**Affiliations:** 1 Innate Immunity Research Lab, Physiology and Experimental Medicine, PGCRL, The Hospital for Sick Children Research Institute, 686 Bay St, Toronto M5G 0A4, Canada; 2 Laboratory Medicine and Pathobiology, Faculty of Medicine, University of Toronto, Toronto, Canada; 3 Institute of Medical Sciences, Faculty of Medicine, University of Toronto, Toronto, Canada

## Abstract

Neutrophils are short-lived innate immune cells. These cells respond quickly to stimuli, and die within minutes to hours; the relevance of DNA transcription in dying neutrophils remains an enigma for several decades. Here we show that the transcriptional activity reflects the degree of DNA decondensation occurring in both NADPH oxidase 2 (Nox)-dependent and Nox-independent neutrophil extracellular trap (NET) formation or NETosis. Transcriptomics analyses show that transcription starts at multiple loci in all chromosomes earlier in the rapid Nox-independent NETosis (induced by calcium ionophore A23187) than Nox-dependent NETosis (induced by PMA). NETosis-specific kinase cascades differentially activate transcription of different sets of genes. Inhibitors of transcription, but not translation, suppress both types of NETosis. In particular, promoter melting step is important to drive NETosis (induced by PMA, *E. coli* LPS, A23187, *Streptomyces conglobatus* ionomycin). Extensive citrullination of histones in multiple loci occurs only during calcium-mediated NETosis, suggesting that citrullination of histone contributes to the rapid DNA decondensation seen in Nox-independent NETosis. Furthermore, blocking transcription suppresses both types of NETosis, without affecting the reactive oxygen species production that is necessary for antimicrobial functions. Therefore, we assign a new function for transcription in neutrophils: Transcriptional firing, regulated by NETosis-specific kinases, helps to drive NETosis.

Neutrophils are terminally differentiated innate immune cells that have highly condensed and uniquely multi-lobulated nuclei. In 1996, Takei *et al*. reported an unusual form of cell death, in which dying neutrophils release chromatin^1^. This novel form of phorbol myristate acetate (PMA)-induced neutrophil death is different than apoptosis and necrosis. About a decade after, in 2004, a similar form of cell death with the release of neutrophil extracellular traps (NETs) was reported during bacterial infection^2^. This form of neutrophil death was defined as a novel form of programmed cell death, NETosis, in 2007^3^. Extensive studies conducted over the last decade established that not only PMA but also various bacteria and bacterial components, including LPS, activate NADPH oxidase 2 (Nox)-dependent NETosis^4^,^5^,^6^,^7^,^8^. Several studies show that extracellular signal-related kinase (Erk), a mitogen-activated protein kinase (MAPK), is one of the major kinases important for Nox-dependent NETosis^4^,^9^,^10^,^11^. Our recent studies show that another kinase, Akt, is also important for Nox-dependent NETosis, and inhibitors of Erk or Akt significantly suppress PMA-mediated NETosis^9^,^10^. Nevertheless, it remains to be determined how these kinases participate in Nox-dependent NETosis.

The second major type of NETosis does not require Nox activation. This Nox-independent NETosis requires increase in intracellular calcium concentration (e.g., induced by calcium ionophores A32178, and ionomycin secreted by Gram-positive bacteria *Streptomyces conglobatus*)^10^,^12^,^13^,^14^. When cytoplasmic calcium level increases, peptidylarginine deiminase 4 (PAD4 or PADI4) constitutively present in the neutrophil cytosol forms a complex with calcium, and rapidly translocates into the nuclei^12^,^13^. PAD4 deiminates positively charged arginine present on histones into non-charged citrulline^13^,^15^,^16^. Therefore, the citrullination of histones is considered to be the driver of DNA decondensation and NETosis^15^,^17^,^18^. However, we have recently shown that citrullination of histone is a hallmark of Nox-independent NETosis (e.g., A32178-mediated NETosis), but not Nox-dependent NETosis (e.g., PMA-mediated NETosis)^10^. These studies also show that Akt, but not Erk, is highly activated during Nox-independent NETosis, and Akt inhibitors significantly suppress such NETosis^9^,^10^. Once again, the involvement of Akt and other kinases in executing calcium-mediated Nox-independent NETosis has also not been clearly established.

In this study, we considered that the process of transcription itself is important to drive NETosis, and specific transcriptional steps could contribute to the decondensation of chromatin required for NETosis. Here, we tested the hypothesis that transcriptional firing, regulated by specific sets of kinases, helps to drive both types of NETosis via active chromatin decondensation. In particular, we aimed to test whether neutrophils differentially transcribe different sets of genes during Nox-dependent and -independent NETosis at different time points. We also examined whether specific kinases that regulate different sets of transcription factors could facilitate transcriptional firing in multiple loci of chromosomes to promote chromatin decondensation. To answer these questions, we used the prototypic agonists PMA and A23187 to induce Nox-dependent and Nox-independent NETosis, respectively. We then conducted transcriptomics studies, and used translation and transcription inhibitors to identify potential molecular steps necessary for driving NETosis. We also confirmed the importance of transcription for biologically relevant agonist-induced NETosis (LPS and ionomycin to induce Nox-dependent and calcium-mediated Nox-independent NETosis, respectively). This new concept - that transcription helps to drive NETosis - could greatly facilitate our understanding of molecular mechanisms governing NETosis.

## Results

### Transcriptional activity reflects degree of DNA decondensation during NETosis

To determine the relevance of transcription in NETosis, we first induced NETosis in human neutrophils, imaged the cells and quantified the morphological changes in nuclear decondensation. Confocal microscopy images show that purified neutrophils maintained clearly defined polymorphonuclear morphology typical of unactivated neutrophils (negative controls; Supplementary Fig. S1A). Qualitative examination shows that at the 30-min time point more neutrophil nuclei delobulated and decondensed in A23187-mediated NETosis compared to PMA-mediated NETosis. However, at the 60-min time point, most of the neutrophil nuclei in both PMA-mediated and A23187-mediated NETosis were decondensed. Some NETs were also visible in A23187-mediated NETosis at this time point. Counting NETotic neutrophils present in each condition confirmed that neutrophil nuclei decondense more rapidly during A23187-mediated NETosis than PMA-mediated NETosis (Supplementary Fig. S1B).

To determine the relevance of transcriptional activity during NETosis in the same time period, we extracted total RNA from neutrophils at 30- and 60-min time points, and conducted a genome-wide transcriptomics analysis of ~67,500 different transcript clusters (genes). These transcript clusters represented both protein coding and non-coding regions of DNA. We standardized the expression values of each transcript in NETotic neutrophils at 30- and 60-min time points. We next validated the quantitative nature of our transcriptomics data by quantitative PCR (q-PCR) using cDNA and specific primers designed to represent the up-regulated genes at both of these two time points (Supplementary Fig. S2, Table S1). The results show that microarray data sets are highly quantitative.

**Figure 1:**
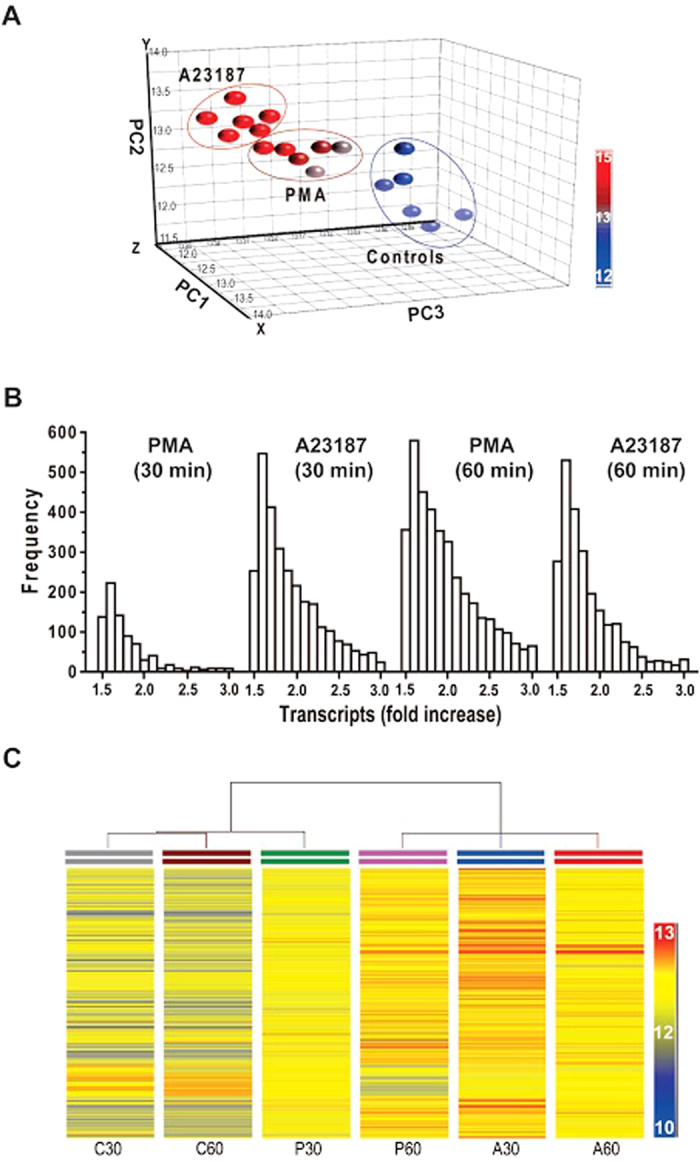
Degree of transcription reflects the extent of nuclear decondensation during NETosis. Transcriptomics was conducted for control, PMA and A23187 conditions at 30- and 60-min time points (n = 3 for each condition). (**A**) Principal component analyses of the transcriptomics data set show that the expression profiles of all 6 conditions are different from each other. Each small ball represents one replicate. (**B**) Frequency distribution of transcripts shows that different degree of transcription occurs under all four different conditions (Chi-square test, p < 0.05). Note that the magnitude of frequencies reflects the level of transcription in each condition. (**C**) Hierarchical cluster analyses of the transcriptomics data show the relatedness of each data set. See Supplementary Fig. S1 for nuclear decondensation during NETosis.

To evaluate the degree of differences and relatedness of transcription profiles among different conditions, we conducted principal component analysis (PCA). PCA clustered the multidimensional data into assigned sets of variables to show transcription profile patterns in each condition. These analyses show that transcriptomes of unstimulated, PMA-activated and A23187-activated neutrophils form distinct clusters (Fig. 1A). Analyses of transcript that show any increase above baseline showed the expression of a large number of transcripts: for PMA, 14602 (22%) at 30 min, 21927 (33%) at 60 min, and for A23187, 27767 (41%) at 30 min and 28914 (43%) at 60 min. Considering the transcripts with ≥1.5-fold increase (a commonly used cut off) as a proxy for transcription at different loci, we calculated frequency distributions, which are significantly different from each other. These analyses indicate that active transcription occurs in more loci in A23187-treated cells than in PMA-treated cells, particularly at the 30-min time point (Fig. 1B). At the later time point (60 min), transcription occurs in many loci in both PMA- and A23187-treated neutrophils.

Hierarchical clustering analysis further shows the overall relatedness of gene expression profiles of these groups (Fig. 1C): control samples have a basal level of transcription, and the transcription in PMA-induced neutrophils at 30-min time point is distinct from the unstimulated controls. Because of the relatively low level of transcriptional activation at 30-min time point in PMA-mediated NETosis, this group clusters next to baseline controls. Transcription in PMA-activated neutrophils at 60-min time point clusters with that of A23187-activated neutrophils, suggesting increased level of transcription is taking place in Nox-dependent NETosis similar to that of Nox-independent NETosis at this time point. Collectively (Fig. 1; Supplementary Figs S1 and S2), these data suggest that transcriptional activity reflects the degree of DNA decondensation occurring during different time points and types of NETosis.

### Transcriptional firing occurs at multiple loci throughout the genome during NETosis

**Figure 2:**
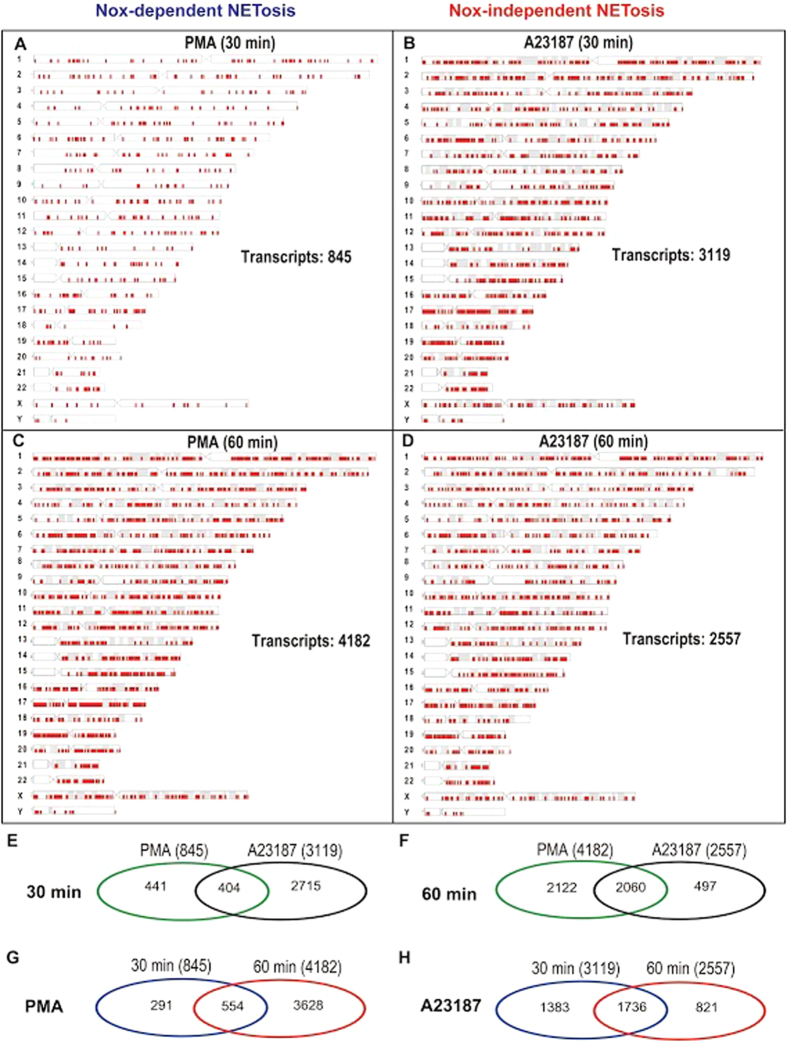
Genome-wide transcription occurs during both Nox-dependent and -independent NETosis. (**A**–**D**) Locations of transcripts, that show 1.5-fold increase, are indicated with red tick marks along each chromosome. Loci of both coding and non-coding transcripts are shown. (**E**–**H**) Venn diagrams showing the numbers of common and unique transcripts detected during PMA- and A23187-mediated NETosis at 30- and 60-min time points. See Supplementary Fig. S3 for percentages.

To verify whether transcriptional firing occurs throughout the genome, we plotted the loci that show ≥1.5-fold increase in transcription on each chromosome (Fig. 2). These maps include loci for coding and non-coding transcripts. At the 30-min time point, 3.69-fold more loci are transcribed in A23187-mediated NETosis compared to PMA-mediated NETosis (Fig. 2A,B). At the 60-min time point, large numbers of loci are transcribed in both PMA-mediated and A23187-mediated NETosis (Fig. 2C,D). Notably, the loci of transcriptions are distributed throughout the entire genome during both types of NETosis at both time points (Fig. 2A–D). Therefore, transcription occurs in multiple loci in all the chromosomes during both types of NETosis.

### Similar as well as distinct loci are transcribed during Nox-dependent and Nox-independent NETosis

Because DNA decondensation occurs differentially in Nox-dependent and -independent NETosis, we asked whether the same or different sets of loci are transcribed in neutrophils treated with two types of NETosis-inducing agonists. Venn analysis shows that approximately half of the loci transcribed in PMA-treated cells were transcribed in A23187-treated cells at both time points (Fig. 2E,F; Supplementary Fig. S3). Transcriptional activity is low at the 30-min time point in PMA-treated cells. However, transcription during both types of NETosis was high at the 60-min time point, and 80.5% of the loci transcribed in A23187-treated were also transcribed in PMA-treated neutrophils at this time point.

In PMA-mediated NETosis, approximately two third of the loci transcribed (≥1.5-fold increase) at the 30-min time point were also transcribed at the 60-min time point. Many more loci are transcribed at the 60-min time point, and hence, 86.8% of the transcribed loci at this time point were different than those transcribed at the 30-min time point (Fig. 2G; Supplementary Fig. S3). In A23187-treated cells, approximately half of the loci transcribed at the 30-min time point were also transcribed at the 60-min time point. About a third of the loci transcribed at the 60-min time point were different than those at the 30-min time point in A23187-treated cells (Fig. 2H; Supplementary Fig. S3). Therefore, (i) chromosome-wide transcription occurs at multiple loci during NETosis, and (ii) transcription overlaps in many, but not in all, loci at different time points in both of these types of NETosis.

### Different sets of coding genes are transcribed during Nox-dependent and -independent NETosis

**Figure 3:**
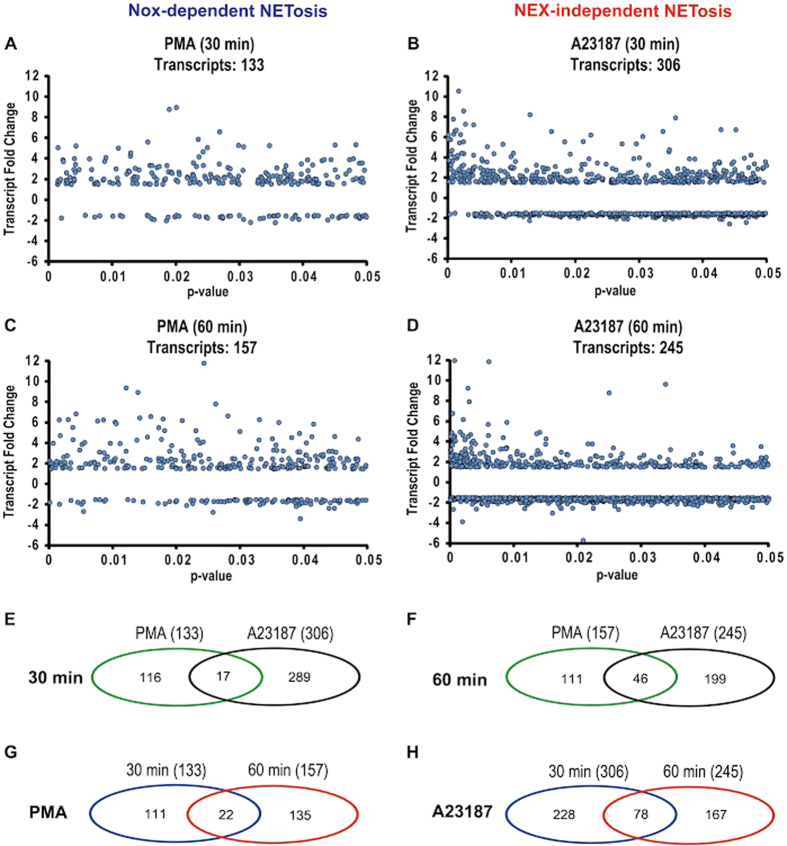
Different sets of coding genes are expressed differentially during Nox-dependent and -independent NETosis. (**A**–**D**) Probability distributions of highly expressed coding genes (1.5-fold difference). (**E**–**H**) Venn diagrams show the numbers of common and unique coding genes with p < 0.05 that are transcribed in PMA mediated and A23187-mediated NETosis at 30- and 60-min time points. See Supplementary Fig. S4 for percentages and Table S2 for a complete gene list.

We next focused on identifying whether unique coding genes (≥1.5-fold difference with p < 0.05) are differentially expressed in both of these types of NETosis. Probability distribution plots and Venn diagrams show that mostly different genes were expressed at different stages and types of NETosis (Fig. 3A–D; Supplementary Fig. S4; Supplementary Table S2). In general, distinct sets of genes were transcribed at 30- and 60-min time points, in each condition (Fig. 3E,F; Supplementary Fig. S4). Only 14.0–16.6% of the coding genes transcribed in PMA-treated cells are transcribed at both the 30- and 60-min time points (Fig. 3G; Supplementary Fig. S4). Similarly, A23187-treated neutrophils mainly transcribed distinct sets of genes at these two time points. About a quarter to a third of these genes are expressed at both 30- and 60-min time points (Fig. 3H; Supplementary Fig. S4). These analyses show that different sets of coding genes are transcribed at different time points and in these two types of NETosis. Comparing the transcripts at the peak expression time points (PMA condition at 60 min *vs*. A23187 at 30 min) shows that only 35% and 18% transcripts overlap for PMA (60 min) and A23187 (30 min) conditions, respectively (Supplementary Fig. S4E). Therefore, most of the coding genes show treatment specific (PMA and A23187) transcription.

### Different sets of kinases activate transcription during Nox-dependent and -independent NETosis

**Figure 4:**
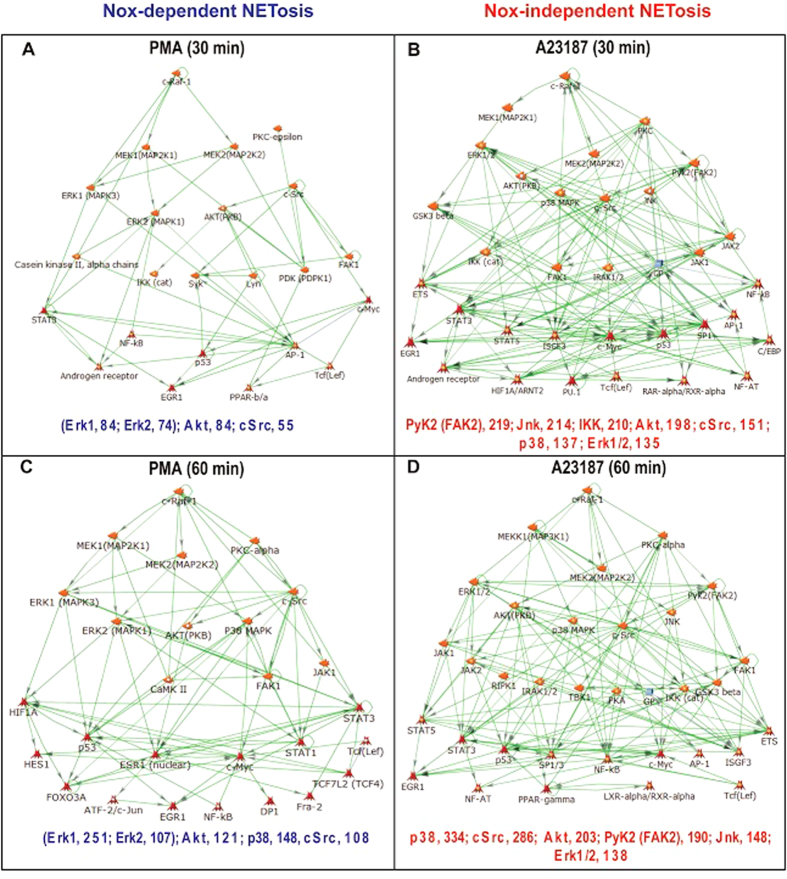
Transcription network analysis indicates that different sets of kinases could drive the transcription during both types of NETosis. (**A**–**D**) Network analysis at 30- and 60-min time points for both PMA-mediated and A23187-mediated NETosis showing the activation of different transcription factors by various kinase cascades. Transcripts used in this analysis include all the coding genes that showed 1.5-fold increase with a p-value of <0.05 shown in Fig. 3 and Table S2. Values at the bottom of the network maps represent the numbers of transcripts predicted to be transcribed by phosphorylation of specific kinase cascades and transcription factors. See Table S3 for transcription factors and number of regulated genes.

Erk, p38 and Akt are differentially activated during Nox-dependent and -independent NETosis^4^,^9^,^10^,^11^,^19^. Therefore, to identify the kinases that could induce transcription during Nox-dependent and -independent NETosis at different time points, we conducted transcriptional regulatory network analyses with coding genes as proxies (≥1.5-fold difference; Fig. 4A–D; Supplementary Table S3). In PMA-mediated NETosis, kinases such as Erk1, Erk2, Akt and cSrc could activate the transcription of 84, 74, 84 and 55 genes, respectively, at the 30-min time point. Combining Erk1 and Erk2 into one group indicates that Erk, Akt, cSrc could activate the transcription of 158, 84 and 55 genes, respectively (Fig. 4A). Similar analysis conducted at the 60-min time point indicates that Erk1, Erk2, Akt, p38 and cSrc could activate the transcription of 251, 107, 121, 148 and 108 genes, respectively. Again, combining different Erks indicates that Erk, Akt, p38 and cSrc could activate the transcription of 358, 121, 148 and 108 genes, respectively (Fig. 4C). Note that certain genes are activated via more than one kinase cascades, resulting in slight differences in the number of genes listed in Figs 3E–H and 4. Overall, these analyses show that Erk, Akt, p38 and cSrc are some of the major kinases that could drive transcription during PMA-mediated Nox-dependent NETosis.

By contrast to PMA-mediated NETosis, A23187-mediated NETosis primarily uses different sets of kinases (Fig. 4B,D). At the 30-min time point, A23187-mediated NETosis uses kinases such as PyK2 (FAK2), Jnk, IKK, Akt, cSrc, p38 and Erk1/2 that can activate the transcription of 219, 214, 210, 198, 151, 137 and 135 genes, respectively (Fig. 4B). At the 60-min time point, some of the same kinases p38, cSrc, Akt, PyK2 (FAK2) Jnk and Erk continue to activate the transcription of 334, 286, 203, 190, 148 and 138 genes, respectively (Fig. 4D). These analyses show that p38, cSrc, Akt, Jnk and Erk1/2 are some of the major kinases that contribute to the transcription of A23187-mediated Nox-independent NETosis.

Collectively, these analyses highlight that a key set of kinases such as Erk, Akt, p38 and cSrc (kinases are listed in the order in which they are responsible for activating the highest to the lowest numbers of genes) primarily drive the transcription during PMA-mediated Nox-dependent NETosis (Fig. 4). By contrast, several kinases including PyK2 (FAK2), Jnk, IKK, Akt, cSrc, p38 and Erk (listed in the order from the highest to the lowest) can drive the transcription during A23187-mediated Nox-independent NETosis. Differences in transcription activation at different time points could be attributable to the activation of different intermediate kinase cascades at early and later time points. This is apparent from the increase in the connectivity of the networks at later time points in both types of NETosis.

### Inhibition of transcription initiation suppresses both PMA- and A23187-induced NETosis

**Figure 5:**
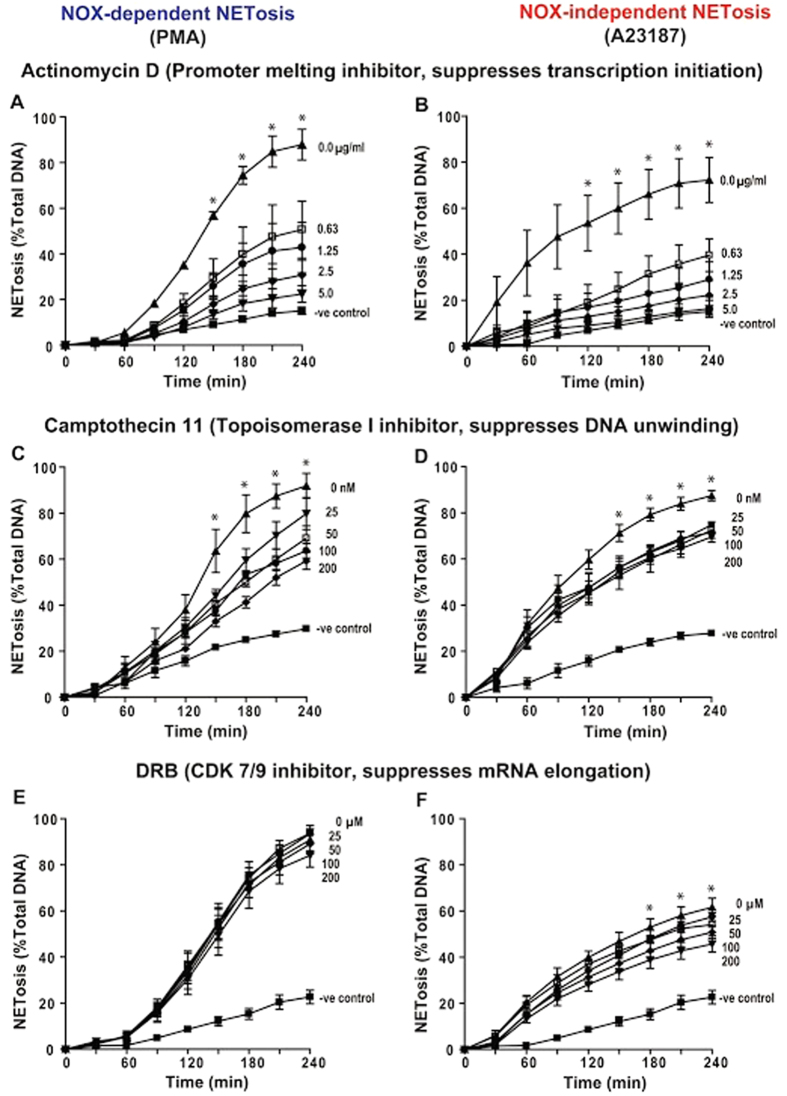
Sytox green assays show that inhibition of initial steps of transcription suppresses both types of NETosis. (**A**) Effect of G-C rich promoter melting inhibitor Actinomycin D (0, 0.625, 1.25, 2.5, 5.0 μg/ml) on PMA-mediated NETosis. (**B**) As of (**A**) but for A23187-mediated NETosis. (**C**) Effect of DNA topoisomerase 1 inhibitor Camptothecin 11 (0, 0.625, 1.25, 2.5, 5.0 nM) on PMA-mediated NETosis. (**D**) As of (**C**) but for A23187-mediated NETosis. (**E**) Effect of mRNA elongation inhibitor, that prevents the phosphorylation of CDK 7 and 9 to limit RNA polymerase movement on DNA (0, 25, 50, 100, 200 μM DRB) on PMA-mediated NETosis. (**F**) As of (**E**) but for A23187-mediated NETosis. *Indicates that NETosis with no inhibitor significantly differs from the rest of the conditions (p < 0.05; One-Way ANOVA with Dunnett’s post-test conducted at each time points; n = 4). See Supplementary Fig. S5 for additional information.

To determine the key steps necessary to drive NETosis, we preincubated neutrophils with various transcription inhibitors, and induced NETosis with PMA or A23187. Actinomycin D (ActD) is a commonly used transcription inhibitor, which preferentially binds to destabilized G-C rich promoter regions of DNA and blocks transcription initiation. Sytox green-based NETosis assays showed that ActD (0–5 μg/ml) dose-dependently inhibited both types of NETosis (Fig. 5A,B; Supplementary Fig. S5A,B). Topoisomerase I inhibitor, Camptochecin 11 also dose-dependently inhibited both types of NETosis to some extent; the effect was more prominent in Nox-dependent NETosis than in Nox-independent NETosis (Fig. 5C,D; Supplementary Fig. S5C,D). The RNA polymerase inhibitor, 5,6-dichloro-1-β-D-ribofuranosyl benzimidazole (DRB), which limits mRNA elongation, did not show significant inhibition of Nox-dependent NETosis; however, DRB inhibited Nox-independent NETosis in a dose-dependent manner, to some extent (Fig. 5E,F; Supplementary Fig. S5E,F). Alpha amanitin that inhibits mRNA elongation did not show significant inhibition of any types of these NETosis (data not shown). These results collectively indicate that initial steps of transcription and unwinding of DNA are more important than full transcription itself for NETosis, although some differences exist between these two types of NETosis.

**Figure 6:**
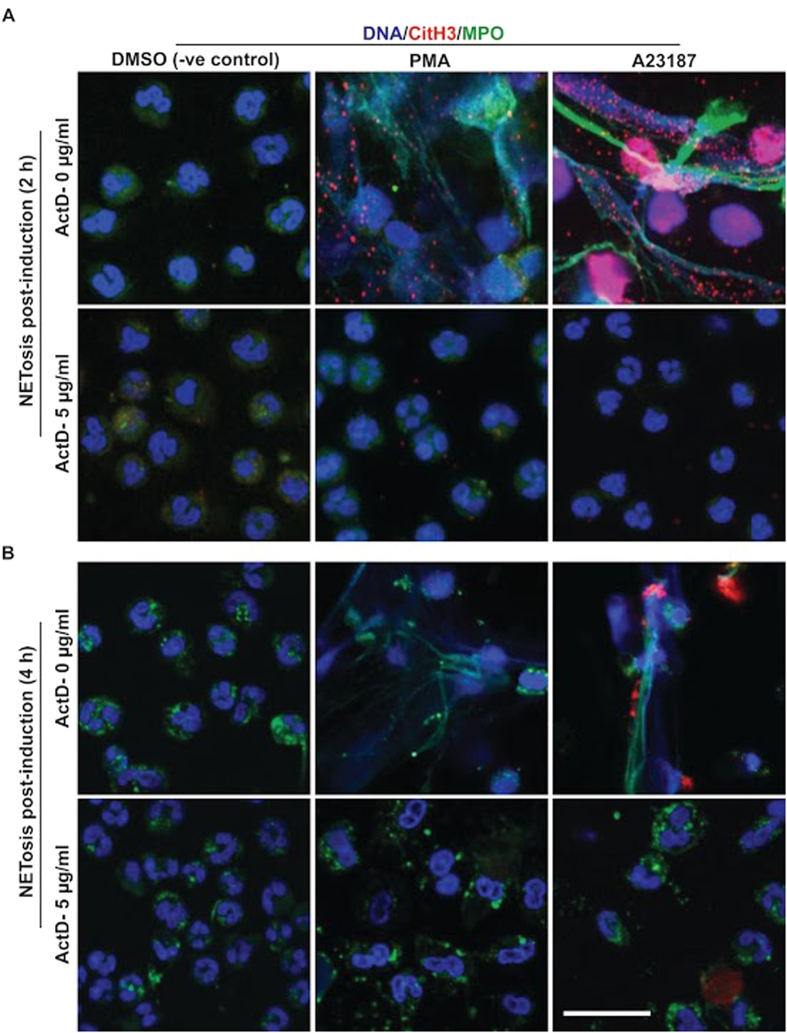
Confocal microscopy images show that transcription inhibitor Actinomycin D suppresses PMA-mediated and A23187-mediated NETosis. (**A**) Unstimulated control neutrophils show typical polymorphonuclear morphology. Myeloperoxidase (MPO) is visible in the cytoplasm. MPO co-localizes to NET DNA generated by PMA-mediated NETosis. A limited amount of citrullinated histone 3 (CitH3) immunostaining is visible on some of the NETs. By contrast, intense immunostaining of CitH3 is visible on decondensed nuclei during A23187-induced NETosis; nicely dispersed immunolabeling of CitH3 is visible on the NETs released from these neutrophils. MPO colocalizes to NETs. (**B**) As of (**A**) but with Actinomycin D (ActD, 5 μg/ml). PMA- and A23187-treated neutrophils did not show NETosis, and the nuclear morphology of these cells remains the same as that of the unstimulated control neutrophils. Nuclear morphology of these cells also do not show signs of substantial apoptosis. Only a limited amount of CitH3 staining is detectable in any of the ActD treated conditions. Blue, DAPI staining for DNA; Green, MPO; Red, CitH3. Scale bar, 25 μm. See Supplementary Fig. S6 for additional information.

To confirm Sytox green-based NETosis data, we conducted immunofluorescence microscopy at 2-h and 4-h time points. Image analyses showed that ActD inhibited NETosis in a dose dependent manner, and that 5 μg/ml concentration of ActD blocked both PMA- and A23187-mediated NETosis (Fig. 6A,B; Supplementary Fig. S6). Myeloperoxidase (MPO) colocalization to DNA confirmed typical NETosis induction by the PMA and A23187. Citrullinated histone 3 (CitH3) immunostaining shows that A23187-mediated Nox-independent, but not PMA-mediated Nox-dependent NETosis, induces extensive citrullination of histone 3. A large amount of CitH3 signal was detected within the decondensed nuclei, and a nice chromatin-wide distribution of CitH3 was visible on fully decondensed NETs released during A23187-induced NETosis. The small amount of CitH3 detected in PMA-mediated NETosis was distributed throughout the NETs, and in a few neutrophil nuclei. We also noted that CitH3 formation was detectable to varying degrees in the presence of lower concentrations of ActD during A23187-mediated NETosis; it limited when 5 μg/ml of ActD prevented DNA decondensation. At 4-h time point, small numbers of cells show limited CitH3 and partial DNA decondensation. Few cells showed DNA condensation, but compared to controls, dramatic changes towards apoptosis was not detected in the presence of ActD (Supplementary Fig. S6). This data set (Fig. 6A) indicates that citrullinating histones could enhance DNA decondensation during Nox-independent NETosis, but preventing transcription initiation by ActD (5 μg/ml) suppresses both types of NETosis.

### Actinomycin D suppresses both LPS- and ionomycin-mediated NETosis

**Figure 7:**
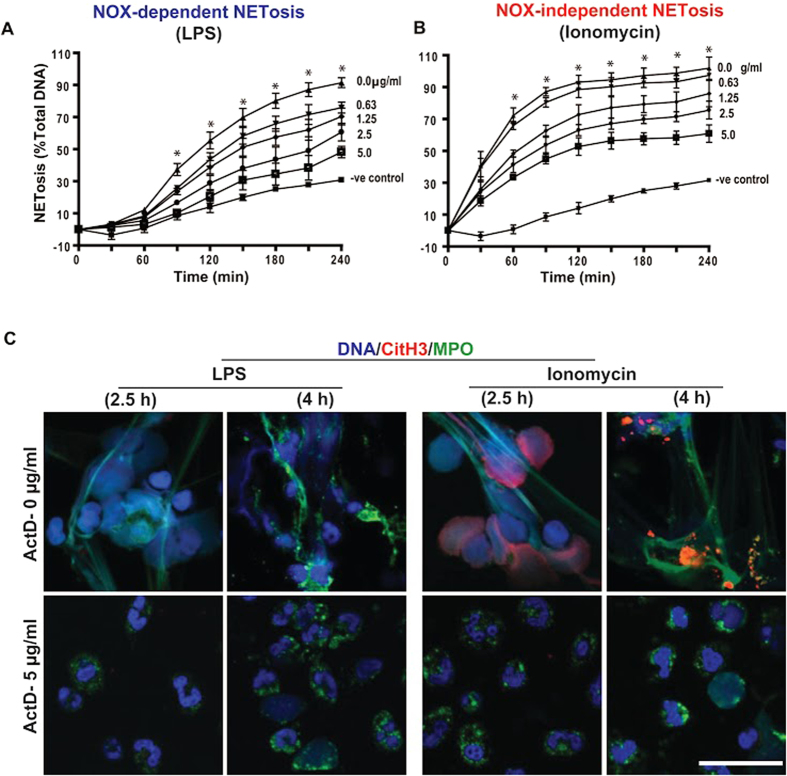
Actinomycin D suppresses NETosis induced by biologically relevant Nox-dependent (LPS) and -independent (ionomycin) agonists. (**A**,**B**) Neutrophils were induced by media control (-ve control), LPS (25 μg/ml) and ionomycin (5 μM) in the presence or absense of ActD (0, 0.625, 1.25, 2.5, 5.0 μg/ml). The Sytox green plate reader data show the suppression of NETosis by ActD in a dose-dependent manner (p < 0.05; One-Way ANOVA with Dunnett’s post-test conducted at each time points; n = 4). (**C**) Confocal imaging of the MPO and CitH3 immunostained cells at 2.5 h show NETosis (NET DNA colocalize with MPO). These ActD treated neutrophils do not show signs of considerable degree of NETosis (nuclear decondensation) or apoptosis (nuclear condensation). Furthermore, ionomycin-mediated NETosis show extensive citrullination of histones (CitH3) on decondensing nuclei, and on the NETs. Blue, DAPI staining for DNA; Green, MPO; Red, CitH3. Scale bar, 25 μm. See Supplementary Fig. S7A and S7B for additional information.

To validate the effect of transcription on NETosis induced by biologically relevant agonists, we used LPS (a Nox-dependent NETosis inducing agonist) and ionomycin (a Nox-independent NETosis inducing agonist). Sytox green assays and confocal imaging show that both LPS and ionomycin induce NETosis with the expected kinetics of (slow) Nox-dependent and (rapid) Nox-independent NETosis, respectively. Sytox green assays show that ActD dose-dependently suppresses NETosis mediated by both of these agonists (Fig. 7A,B; Supplementary Fig. S7). Confocal microscopy confirmed that 5 μg/ml ActD suppresses both LPS- and ionomycin-mediated NETosis at 2.5-h and 4-h time points (Fig. 7C, Supplementary Fig. S7). These images also show that ionomycin, but not LPS, induces high degree of citrullination of histone, and that ActD suppresses citullination. Occasional CitH3 formation and partial chromatin decondensation are detectable in some fields of views (Supplementary Fig. S7). Overall, blocking transcription also suppresses biologically relevant agonist-induced Nox-dependent and Nox-independent NETosis.

### Inhibition of transcription does not affect reactive oxygen species (ROS) generation in neutrophils

ROS production is important for direct anti-microbial functions of neutrophils and NETosis^1^,^4^,^5^,^6^,^9^,^10^,^11^,^20^,^21^. Therefore, we tested whether ActD inhibits ROS production that is necessary for NETosis. ActD did not inhibit PMA-mediated Nox-dependent ROS production measured by DHR123, and A23187-mediated Nox-independent ROS production measured by MitoSox (p > 0.05). This data set further indicates that ActD-mediated suppression of transcription is downstream of ROS production, and hence, transcription-based suppression of NETosis inhibits both types of NETosis without affecting ROS production that is required for antimicrobial functions of neutrophils.

### Translation is not necessary for Nox-dependent and Nox-independent NETosis

A previous study shows that the inhibition of translation does not affect Nox-dependent NETosis induced by LPS^12^. To confirm the relevance of new protein synthesis to NETosis, we induced Nox-dependent NETosis by another agonist, PMA, in the presence of cycloheximide (CHX). The translation inhibitor CHX did not inhibit PMA-induced NETosis, confirming that Nox-dependent NETosis does not require translation of mRNA to protein (Supplementary Fig. S8A). In addition, CHX did not inhibit NETosis induced by A23187, indicating that translation is also not necessary for Nox-independent NETosis (Supplementary Fig. S8B). Another translation inhibitor, Puromycin, also did not inhibit either of these types of NETosis (Supplementary Fig. S8A,B). Taken together, these data indicate that new protein synthesis is neither necessary for PMA-induced Nox-dependent, nor for A23187-induced Nox-independent NETosis.

## Discussion

**Figure 8:**
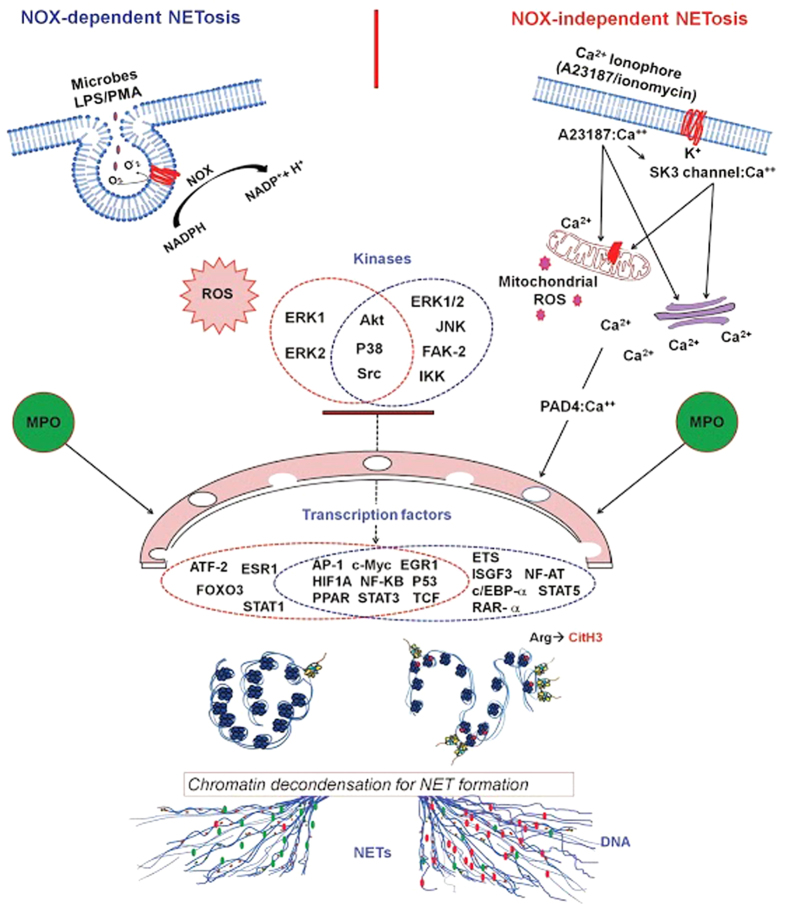
Transcriptional firing model of NETosis. Upon activation with appropriate agonists, neutrophils generate reactive oxygen species, and activate distinct kinase cascades during the early steps of NETosis. Some of the kinases in these cascades phosphorylate and activate specific sets of transcription factors, which help to initiate transcription at specific promoters. Citrullination of histone (CitH3) at promoter regions could help to extensively decondense chromatin, particularly in rapid Nox-independent (intracellular calcium-dependent) NETosis. Genome-wide ranscriptional firing at these loci helps to promote chromatin decondensation necessary for NETosis. During the progression of NETosis, myeloperoxidase (MPO) and other granular components (e.g., elastases and other proteases that cleave chromatin) are deposited on chromatin, which is released as NETs.

Although several key elements of NETosis have been described, the mechanistic details of NETosis have not been fully elucidated. We considered that transcriptional firing at multiple locations of the chromosome could help to drive the decondensation step necessary for NETosis. Our transcriptomics analysis in neutrophils shows that the level of transcription reflects the degree of NETosis, and transcription is initiated much more rapidly in Nox-independent NETosis than Nox-dependent NETosis. Transcriptomics network analysis further shows that NETosis type-specific kinases regulate the transcription in many loci of the chromosomes. Therefore, we propose that transcription-related DNA decondensation is a key factor that helps to drive NETosis (Fig. 8). Pathway analysis of the transcripts suggests that inflammation-related genes are transcribed during different types of NETosis, but these proteins are not necessary for NETosis. Furthermore, citrullination of histone is not sufficient for NETosis, and blocking transcription could block NETosis while maintaining ROS production by neutrophils. This intriguing new concept could help to fully understand the mechanism of NETosis and NETosis-related inflammation.

Neutrophils are short-lived cells, and carry several cytotoxic proteins and enzymes in cytosol and granules^22^,^23^. These pre-packaged neutrophil proteins, but not newly synthesized proteins, have been considered to be important for many known biological functions (e.g., phagocytosis, bacterial killing, apoptosis)^22^,^24^. Why neutrophils transcribe their genomes, particularly during cell death has been an interesting paradox for many decades. We set out to identify whether transcription plays an important biological role during NETosis. It has been well established that Nox-dependent NETosis requires 2–4 hours^5^,^8^,^9^,^10^,^11^. During NETosis, multi-lobulated polymorphonuclei gradually decondense before the NETs are released^5^,^25^,^26^,^27^. At the 30- and 60-min time points, most of the cells are not permeable to Sytox green. Sytox green readings and manually quantifying nuclear morphology seen in our study are consistent with these facts (Figs 1, 5, 6 and 7). Our recent studies show that Nox-independent NETosis, induced by A23187 and ionomycin, occurs rapidly, and reaches maximal level within 1–2 hours^10^. Certain strains of *S. aureus* that secrete Penton-Velentine toxin induce Nox-independent NETosis within 15 min^28^. At the end of NETosis, most of the cells are essentially dead, although enucleated cytoplasts could remain functional for some time^29^,^30^, and significant amounts of the nucleic acids are released into the extracellular environments^31^,^32^. RNA is a highly labile molecule, easily degraded by the RNAse enzymes ubiquitously present in cells. Therefore, we have limited our transcriptomics analysis to the maximum time point of 60-min. Data obtained in this study is consistent with rapid nuclear decondensation occurring in Nox-independent NETosis; and transcription levels reflect the degree of nuclear decondensation in both types of NETosis (Fig. 1; Supplementary Fig. S1).

The transcriptomics array chip used in this study had the ability to detect ~67,500 transcript clusters. These probes covered most of the genome. Large numbers of genes are differentially transcribed across the genome of neutrophils^33^,^34^,^35^,^36^,^37^,^38^. Analysis of transcripts that are upregulated during the induction of NETosis showed that transcription occurs at multiple loci of the chromosomes in both types of NETosis, and numbers of transcripts increased with time (Figs 1 and 2; the number of genes would depend on the cut off points; although large numbers of genes are transcribed during NETosis, we used the conventional 1.5-fold cut off as a proxy for transcription in most of our analyses). Transcriptomic analysis of neutrophils after inducing NETosis with bacteria is technically challenging because of the presence of large amounts of bacterial mRNA that could hybridize to homologous genes in the human microarray. Therefore, we have limited our transcriptomics analysis and conclusion to prototypical agonists of NETosis for this proof of principal study.

Different kinases are mainly activated during different types of NETosis (e.g., Erk, p38, PKC, PI3K, Akt, cSrc)^4^,^9^,^10^,^11^. These kinases could be important for regulating various cellular events in neutrophils. However, their roles in NETosis have not been clearly understood. We considered that one of the major functions of these kinases is to activate specific transcription factors directly, or indirectly via kinase cascades. Transcription activation network analysis indicates that different kinases such as Erk, Akt, p38, and cSrc-regulated genes are primarily transcribed during Nox-dependent NETosis. By contrast, the transcription of Akt, p38, cSrc, PyK2 and Jnk-regulated genes are mainly transcribed in Nox-independent NETosis (Figs 3 and 4). The Raf-Mek-Erk kinase cascade is important for Nox-dependent NETosis, and inhibition of Erk significantly suppresses Nox-dependent NETosis^4^,^9^,^10^,^11^. These are consistent with the increase in Erk-regulated transcripts identified in the network analysis.

The kinases that regulate Nox-independent NETosis have not been fully established. Our recent studies show that Erk is not highly activated during Nox-independent NETosis^10^. Akt is essential for this type of NETosis. However, the activation of p38 has been noted at baseline, and in some studies, but not in others, p38 inhibitors significantly inhibited NETosis^10^,^11^. The network analysis identified a number of kinases that differentially activate transcription in Nox-independent NETosis at different time points. These data, at least in part, explain why Nox-independent NETosis induces high levels of transcription starting from an early time point (30 min); efficient chromatin decondensation takes place rapidly in Nox-independent NETosis compared to Nox-dependent NETosis (Figs 1, 3 and 4). Nevertheless, since these two different types of NETosis can still reach the final goal of decondensing chromatin for NETosis via different sets of transcripts (genes) activated by specific transcription factors, the process of transcription in multiple loci, but not specific genes, are important for driving NETosis.

Our recent studies also show that extensive citrullination of histone occurs during Nox-independent NETosis, but not in Nox-dependent NETosis^10^. Citrullination of histone at promoter sites is known to provide access to transcription factors^12^,^13^,^39^,^40^,^41^. Confocal microscopy images show that vast amounts of CitH3 are nicely distributed throughout the NETs formed during calcium-mediated Nox-independent NETosis induced by both A23187 and ionomycin (Figs 6 and 7). Therefore, a combined effect of citrullination of histones during calcium-mediated NETosis and kinase-specific transcriptional activation in these loci would lead to the rapid Nox-independent NETosis.

Consistent with our hypothesis, a global transcription inhibitor, Actinomycin D, suppressed both types of NETosis in a dose-dependent manner (Figs 5, 6 and 7). ActD is a highly hygroscopic powder (crystals) and unstable in solution during storage. Therefore, we have conducted serial dilutions of different batches of ActD to reduce potential technical errors. This compound has concentration-dependent effects on different types of RNA polymerases, but at 5 μg/ml ActD suppresses all types of RNA polymerases. Sytox green-based NETosis assay and microscopy images show that suppression is virtually complete, and the cells look similar to control neutrophils in the presence of 5 μg/ml of ActD. Topoisomerase I inhibitor that is known to suppress transcription, particularly from active promoters^42^,^43^,^44^ also showed a partial NETosis inhibitory effect. Recent studies show that release of torsional strain, generated at the promoter regions of DNA, specifically by Topoisomerase I is important for efficient transcription initiation^44^,^45^. By contrast, DRB inhibits specific kinases (e.g., CDK 7 and 9 that activate transcription elongation factor TEFb) that are necessary for facilitating transcription elongation^46^,^47^ partially inhibited Nox-independent NETosis. Some of the differences in degree of inhibition could be related to the CitH3-mediated facilitation of promoter melting and mode of action of the inhibitors. Alpha amanitin binds directly to RNA polymerase and inhibits mRNA elongation. This compounds does not inhibit neither PMA- nor A23187-mediated NETosis, therefore, the elongation event is not essential for NETosis. Overall, these results are consistent with the idea that transcription initiation, but not full transcription of genes per se, helps to drive both types of NETosis.

Transcriptomics studies conducted with LPS-treated neutrophils (an agonist of Nox-dependent NETosis) also show the upregulation of large numbers of genes^48^,^49^. To extend the relevance of transcription to biologically relevant agonist-induced NETosis, we induced NETosis with LPS from Gram-negative bacteria *E. coli* and ionomycin form Gram-positive bacteria *Streptomyces conglobatus*. Sytox green assays and confocal images show that ActD significantly suppresses both LPS- and ionomycin-mediated NETosis (Fig. 7; Supplementary Figs S6 and S7). Our data show a clear dose-dependent (0.63, 1.25, 2.5 and 5 μg/ml) suppression by ActD of both Nox-dependent (induced by PMA and LPS) and -independent (induced by A23187 and ionomycin) NETosis. ActD at 1 μg/ml concentration is known to block the formation of full-length transcripts that are necessary for new protein synthesis. In our studies, 1 μg/ml of ActD partially inhibits NETosis. A recent study examined the effect of ActD at 1 μg/ml on PMA- and Candida albicans-mediated NETosis. and concluded that new protein synthesis is not necessary for NETosis^50^. Therefore, suppressing promoter melting, but not full transcription, is needed for the supression of NETosis.

A previous study conducted by Neeli *et al*. in 2008 showed that translation is not required for LPS-mediated NETosis^12^. Our studies further confirmed that translation is not necessary for Nox-dependent NETosis induced by different agonists. We also show that CHX, which inhibits the translocation step in the protein synthesis by interfering with tRNA and mRNA molecules in the ribosome^51^, did not inhibit A23187-mediated Nox-independent NETosis. Puromycin, which resembles the end of the aminoacylated tRNA, causes premature chain termination during translation in the ribosome^51^, also did not inhibit both types of NETosis. Because both types of inhibitors did not suppress NETosis (Supplementary Fig. S8), we consider that translation is not necessary for any of these two types of NETosis. Furthermore, transcription and subsequent translation typically requires more than 2 hours; therefore, new protein synthesis would not play a significant role in NETosis. Considering the fact that many rapid biological processes do not require new protein synthesis^22^, it is not surprising that translation is not important for NETosis.

Cells that do not typically die by NETosis can also respond to PMA, A23187 and microbial components in various ways. These cells are very different from neutrophils in terms of their cellular content (e.g., neutrophil elastase, proteinase 3, MPO, other granular proteins, PAD4, Nox), and their ability to produce ROS, regulate specific kinases/phosphatases, transcribe specific sets of genes and successfully translate mRNA to generate regulatory proteins. Gene expression in different cell types is tightly controlled by specific sets of kinases and transcription factors. These are potential factors that determine the cell fate and functionality in different cells – activation, differentiation, increased survival, apoptosis, NETosis, phagocytosis, and etc. ROS production is necessary for antimicrobial functions of neutrophils^22^,^52^, and is not inhibited by inhibiting transcription and NETosis. Therefore, some of these nucleic acid metabolism inhibitors could prevent both types of NETosis while maintaining antimicrobial functions in neutrophils. Collectively, these studies (Figs 1, 2, 3, 4, 5, 6, 7, 8; Supplemental Materials) show the relevance of transcription to NETosis, and attribute, at least, one reason why dying neutrophils actively transcribe their genome. This is an interesting example of how appropriately activated neutrophils use their rapid transcriptional firing to achieve their suicidal death to form NETs.

## Materials and Methods

### Research ethics board approval

The study protocol was approved by the Hospital for Sick Children ethics committee, and the informed consents were obtained from the study participants. All methods were performed in accordance with the relevant guidelines and regulations.

### Buffers and chemicals

All buffer salts, inhibitors and other reagents were purchased from Sigma-Aldrich unless otherwise stated.

### Human peripheral neutrophil isolation

Neutrophils from healthy male donors were used in the study. Donors with eosinophils, or either too low or too high neutrophil count were excluded from the study. Typically, our isolation procedure yields 1–2 million neutrophils per ml of blood. Blood from donors that fall outside of this range has not been used in the study. Typically, neutrophils were purified from 40–60 ml of peripheral blood using PolymorphPrep^TM^ (Axis-Shield) according to the manufacturer’s instructions with minor modifications. An equal volume of blood and PloymorphPrep were centrifuged at 600 × g for 35 min with no brakes to get a distinct neutrophil band. Then neutrophils were washed and the residual red blood cells were eliminated by hypotonic hemolysis (0.2% (w/v) NaCl, 30 sec; followed by the addition of an equal volume of 1.6% (w/v) NaCl with 20 mM Hepes; 400 × g, 10 min). Purified neutrophils were re-suspended in RPMI 1640 medium (Invitrogen) supplemented with 10 mM Hepes buffer^9^,^10^,^20^. Cell density of purified neutrophils was quantified using a hemocytometer, and the purity of the neutrophils was determined by Cytospin preparations. Neutrophil preparations with >95–98% were used in all the experiments, reported in this study.

### Sytox green NETosis assay

For the plate reader NETosis assays, a volume of 100- μl media containing 50,000 neutrophils mixed with 5 μM Sytox green (Life Technologies), a cell-impermeable nucleic acid dye, was seeded in 96-well black clear-bottom plates. The indicated concentrations of specific inhibitors (Transcription initiation inhibitor Actinomycin D, 0–5 μg/ml; Topoisomerase I inhibitor Camptothecin 11, 0–200 nM, Tocris Bioscience; CDK 7/9 kinase inhibitor DRB, which inhibits mRNA elongation, 0–200 μM) were added to respective wells with controls (no inhibitors). After 1 h, 5 μl agonists (final concentrations: PMA, 25 nM; A23187, 4 μM; LPS (from *E. coli* O111:B4), 25 μg/ml; ionomycin, 5 μM; −ve control, buffer with 0.4% (v/v) DMSO, which gives an equivalent amount of DMSO present in the agonists) were added, and the changes in green fluorescence signal was measured every 30 min for up to 4 h using a fluorescence plate reader (504 nm excitation, 523 nm emission, POLARstar OMEGA, BMG Labtech). Total DNA (100% DNA) present in these neutrophils was determined by the fluorescence values of the cells lysed with 1% (v/v) Triton-X-100. All the experimental values were standardized to total DNA at each time point.

### Fluorescence confocal imaging

Cells and NETs were fixed with paraformaldehyde (4% (w/v) for 10 min; 2% (w/v) for overnight), and immunostained with various NET markers. Mouse anti-myeloperoxidase antibody (ab25989, Abcam) at 1:500 dilution was used for staining MPO (with secondary antibody conjugated with a green fluorescence Alexa fluor 488 dye; 1:2000 dilution; ThermoFisher Scientific), while rabbit anti-citrullinated histone 3 antibody (ab5103, Abcam) at 1:500 dilution was used for detecting the presence of CitH3 (with secondary antibody conjugated with a far red fluorescence dye Alexa fluor 647; 1:1000 dilution; ThermoFisher Scientific). DNA was stained with DAPI (1:1000 dilution). To obtain high-resolution images, 8-well chamber slides (Falcon culture slides) were used. After completing the immunostaining, the slides were mounted with anti-fade fluorescent mounting medium (Dako) and glass cover slips (Fisher Scientific). True NETosis was confirmed by MPO colocalization to NET DNA by immunofluorescence confocal microscopy (Olympus IX81 inverted fluorescence microscope equipped with a Hamamatsu C9100-13 back-thinned EM-CCD camera and Yokogawa CSU × 1 spinning disk confocal scan head). The confocal images were taken at 40× magnification with 1.35× objective, and processed by Volocity software (version 6.3, Cell Imaging Perkin-Elmer).

### RNA extraction and quality control

Neutrophils (2 × 10^6^ per condition) were incubated for 30 or 60 min in the presence of buffer (RPMI with 10 mM HEPES, PMA (25 nM) or A23187 (4 μM) in Eppendorf tubes at 37 °C in 5% (v/v) CO_2_. After the incubation, cells were collected (1000 × g, 20 min) and frozen at −80 °C with anti-RNase (RNAlater®, which stabilizes and protects RNA by inactivating RNase). Total RNA was isolated from all the stored samples using Trizol (Invitrogen) and RNAEasy Mini kit (Qiagen), following the instructions of manufactures. The yield, integrity and purity of extracted RNA were assessed by Bioanalyzer 2100 and RNA 6000 Nano Lab Chip (Agilent Technologies)^53^. The parallel duplicate tubes of the purified RNA (with RNA integrity number, RIN > 7.5) were placed in 100% ethanol and frozen at −80 °C for transcriptomics experiments.

### Human transcriptome array 2.0 (HTA2) hybridization

Individual RNA samples (each 200 ng) from control, PMA and A23187 conditions at 30- and 60-min time points were used for generating labeled probes for hybridization (3 conditions × 2 time points × 3 donors; 18 samples). We also ensured that the spontaneous background activation in these samples is less than 20% (of total DNA, by Sytox green assay) on the day of RNA collection, to minimize variability. Neutrophils from these three donors responded well to PMA and A23187, and showed 65–90% of NETosis (of total DNA, by Sytox green assay).

These probes were fragmented and hybridized to human transcriptome 2 (HTA 2) Affymetrix gene-chips for 16 h at 45 °C. These chips were washed and scanned using the Affymetix platform at our core facility. HTA2 array chip contains a total of 67528 genes (transcript clusters). It has 44699 protein-coding genes (transcript clusters): these genes are represented by 245349 transcript segments, 560472 exons, 296058 exon clusters and 339146 splice junctions. The chip also has 22829 non-protein coding genes (transcript clusters): these genes are represented by 40914 transcript segments, 109930 exons and 82444 exon clusters. The whole microarray experiments were performed according to the manufacturer’s recommendations and data were generated in compliance with the standard minimum information about a microarray experiment (MIAME) guidelines. We have validated the microarray data using 15 sets of primers: 13 transcripts that showed greater fold-changes in transcription, and 2 unaffected housekeeping gene controls. q-PCR was performed for these 14 segments using the RNA preparations used in the transcriptomics experiments (StepOnePlus, Real-Time PCR, Applied Biosystem). Fold-change analysis was performed by the ΔΔC_t_ method (Supplementary Fig. 2S and Table S1).

### Transcriptome data analysis

Affymetrix .CEL files were imported to Partek Genomics Suite version 6.5 (Partek Inc.) for robust multi-array averaging (RMA) normalization. Per-chip and per-gene intensity dependent normalization (two-way normalization), MA-plot normalization (M, difference between log intensities; A, average log intensity), exploratory analysis, hierarchical clustering analysis and significant ratio analysis were conducted using Transcriptome Analysis Console 3.0 (TAC3) (Affymetrix), Partek Genomics Suite version 6.5 and GeneSpring GX 13.0 software (Agilent Technologies).

To determine the existence of any intrinsic outlier within the data sets, Principal Component Analysis (PCA) was performed. PCA is an unsupervised multivariate analysis tool that clusters the whole data into new sets of linearly correlated variables referred to as principal components (PCs)^54^. Further, to evaluate the expression pattern of each transcript and overall expression relatedness within different groups and sub-groups, unsupervised hierarchical clustering was performed. For this purpose, a standardized Pearson’s uncentered correlation vector with average linkage was used^55^. Sample to sample expressional relationship and visualization in the form of dendrogram and heatmap, respectively, were obtained by hierarchical cluster analysis.

To evaluate the absolute effect in gene expression, the fold change analysis was performed. The fold-change is a measure of the ratio of the expression “signal” between targets and their respective controls “noise” (e.g., P30 *vs*. C30; P60 *vs*. C60; A30 *vs*. C30; A60 *vs*. C60). The transcripts (both coding and non-coding) showing ≥1.5-fold expression were plotted on their Cytoband location in chromosomes (TAC3 software; Chromosome mapping option). Further, we applied the Welch t-test with Benjamini-Hochberg corrections to obtain the p-values. From these analyses, we have generated a list of transcripts that have ≥1.5 fold increase and a p-value of <0.05. Venn analysis was performed to evaluate the uniqueness and commonality between different sets of data.

### Post-hoc networks and pathway analyses

The differentially regulated (≥1.5 increase with p < 0.05) coding transcripts identified in PMA and A23187 at 30- and 60-min time points were used for building kinase cascades, transcription factors and related gene interaction networks using Ingenuity Pathways Analysis (IPA) software (Ingenuity Systems, Redwood City, CA) and GeneGo Metacore software (Thomson Reuters, St. Joseph, MI, USA).

### Other statistical analyses

One-way ANOVA or Two-way ANOVA with F-test, Bonferroni correction and Dunnett’s post-test were used for identifying the differences among different population means. Comparing values between two samples were tested with t-test (two-tailed). Fixed values were compared with samples using one-sample t-test. Chi-square test was used for determining the differences among the frequency distributions of the fold increase in transcripts. Linear regressions, slopes (H_0_ = 0; F test), r^2^ values, other calculations and graphs were made in GraphPad Prism 4, Excel 2010 and Illustrator CS4. A p-value of <0.05 was considered to represent significant differences between conditions.

## Additional Information

**Accession codes**: All the raw data from 18 samples (3 conditions – control, PMA, A23187; 2 time points –30 and 60min; 3 biological replicates – male donors) have been deposited to NCBI’s Gene Expression Omnibus (GEO) data bank with the accession number of GSE80489 (https://www.ncbi.nlm.nih.gov/geo/query/acc.cgi?token=exstkiqanbebdar&acc=GSE80489).

**How to cite this article:** Khan, M. A. and Palaniyar, N. Transcriptional firing helps to drive NETosis. *Sci. Rep.* **7**, 41749; doi: 10.1038/srep41749 (2017).

**Publisher's note:** Springer Nature remains neutral with regard to jurisdictional claims in published maps and institutional affiliations.

## Supplementary Material

Supplementary Figures and Tables
