# Supplementary material for: Transcriptional firing helps to drive NETosis
Source: Sci Rep. 2017 Feb 8;7:41749. doi: 10.1038/srep41749 (PMC5296899; doi:10.1038/srep41749)
Supplement: Supplementary Figures and Tables [file srep41749-s1.pdf]

Supplementary Figures and Tables

Transcriptional firing helps to drive NETosis

Meraj A Khan[1,2] and Nades Palaniyar[1,2,3]

[1]Innate Immunity Research Lab, Physiology and Experimental Medicine, PGCRL, The Hospital for Sick Children Research Institute, 686 Bay St, Toronto, Canada M5G 0A4. [2]Laboratory Medicine and Pathobiology, [3]Institute of Medical Sciences, Faculty of Medicine, University of Toronto, Toronto, Canada.

Correspondence should be addressed to N.P. (nades.palaniyar@sickkids.ca)

Running title: Transcriptional firing drives NETosis

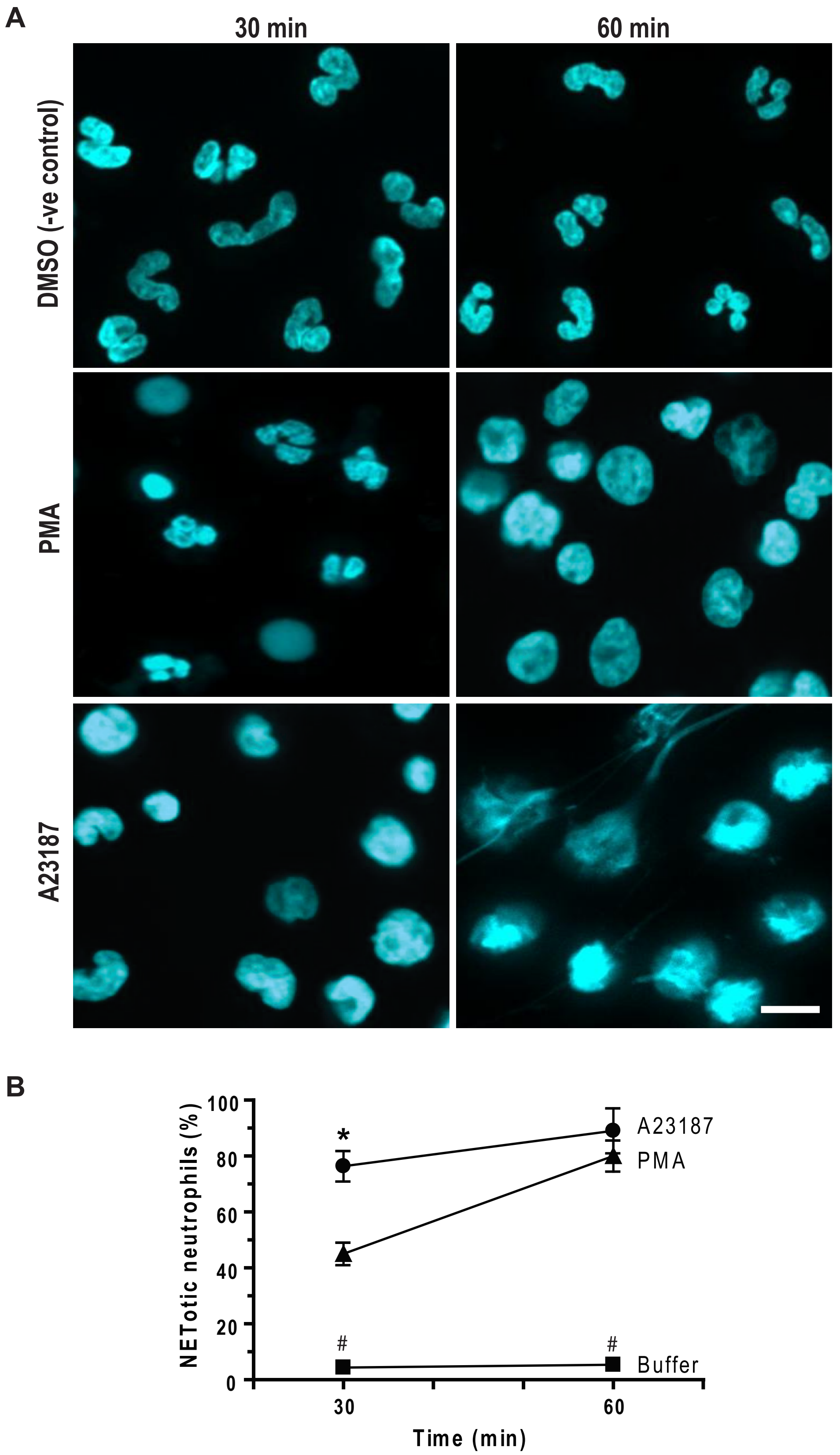

Figure S1. Nuclear decondensation is faster in Nox-independent than Nox-dependent NETosis. (A) Confocal microscopy images show that DNA (blue, DAPI) decondenses rapidly in A23187-mediated Nox-independent NETosis compared with PMA-mediated Nox-dependent NETosis. **(B)** Manual count of the NETotic nuclei (delobulated + fully decondensed + NET releasing). Images from three independent experiments were used; n=74-112 nuclei/condition. * indicates the difference between PMA and A23187 conditions at each time point; # indicates the difference between the agonists (PMA or A23187) from their respective conntrols ($p<0.05$; 2-way ANOVA with Bonferroni post-test).

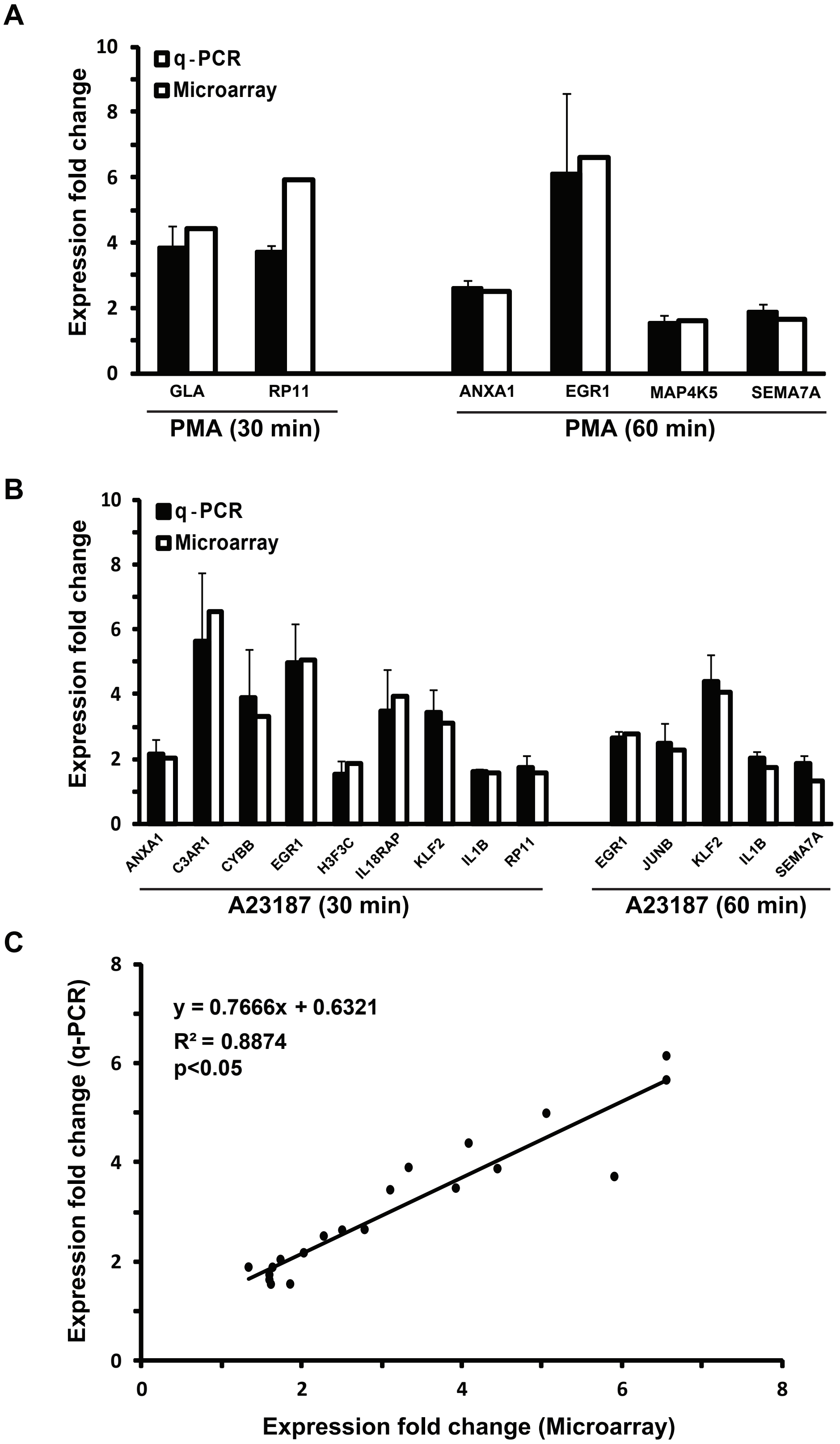

Figure S2. Validation of the expression of selected transcripts by q-PCR. The fold change expression analysis was performed by threshold cycle (ΔΔCt) method. GAPDH (housekeeping gene) was used for standardizing target transcripts. **(A)** The expression profiles of genes obtained by q-PCR (filled bar) and microarray (open bar) in neutrophils treated with PMA at 30- and 60-min time points. **(B)** As of (A), but for A23187 treated cells. **(C)** The linear regression analysis between q-PCR and microarray data for all data points shown in (A) and (B). Details of the primers are given in Supplementary Table S1. (n=3)

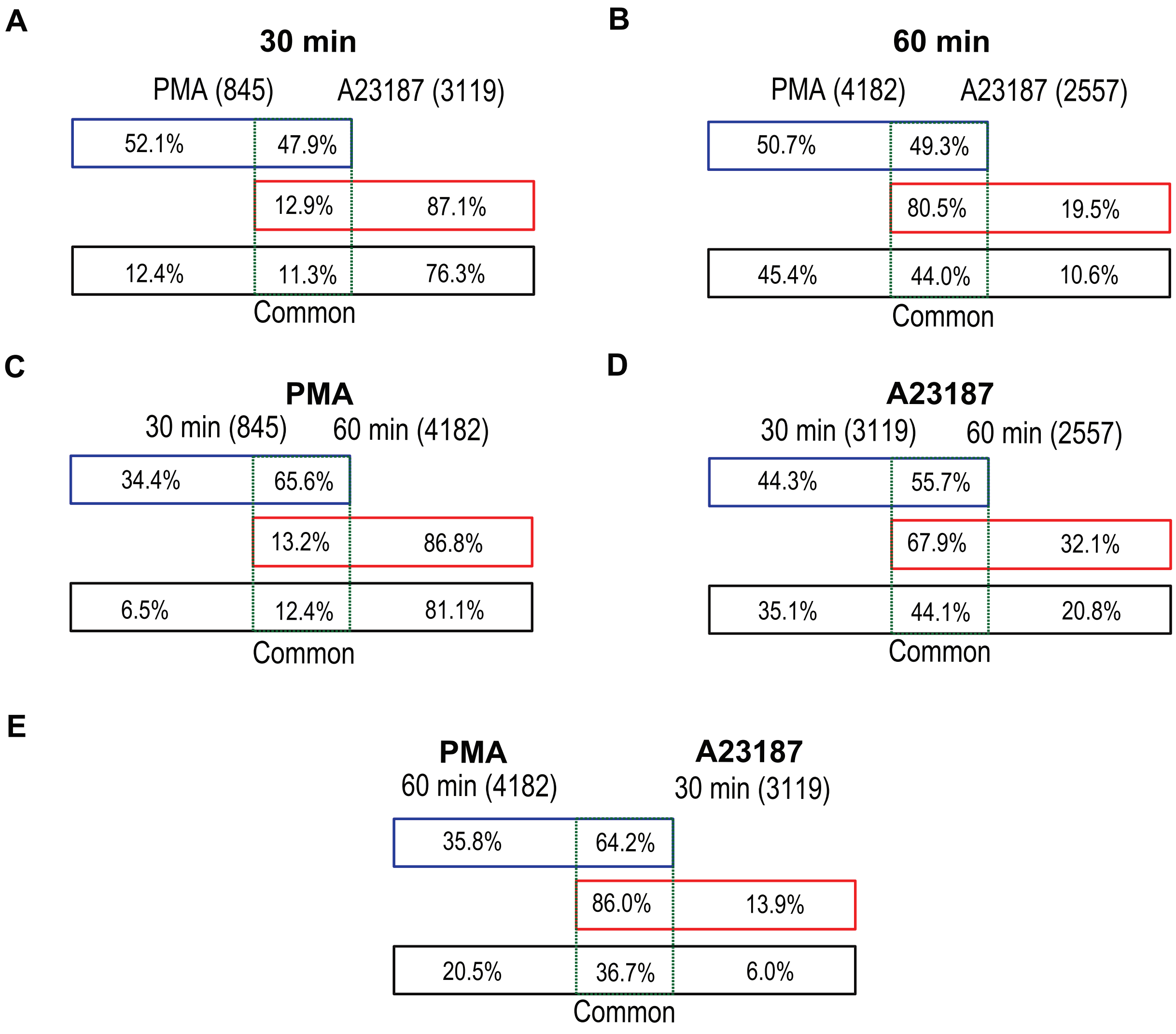

Figure S3. Percentage overlap in transcripts. The percentages of common and unique transcripts (coding and non-coding genes) showing ≥1.5 fold expression have been calculated based on each group, along with the total in both groups (shown in Figure 2).

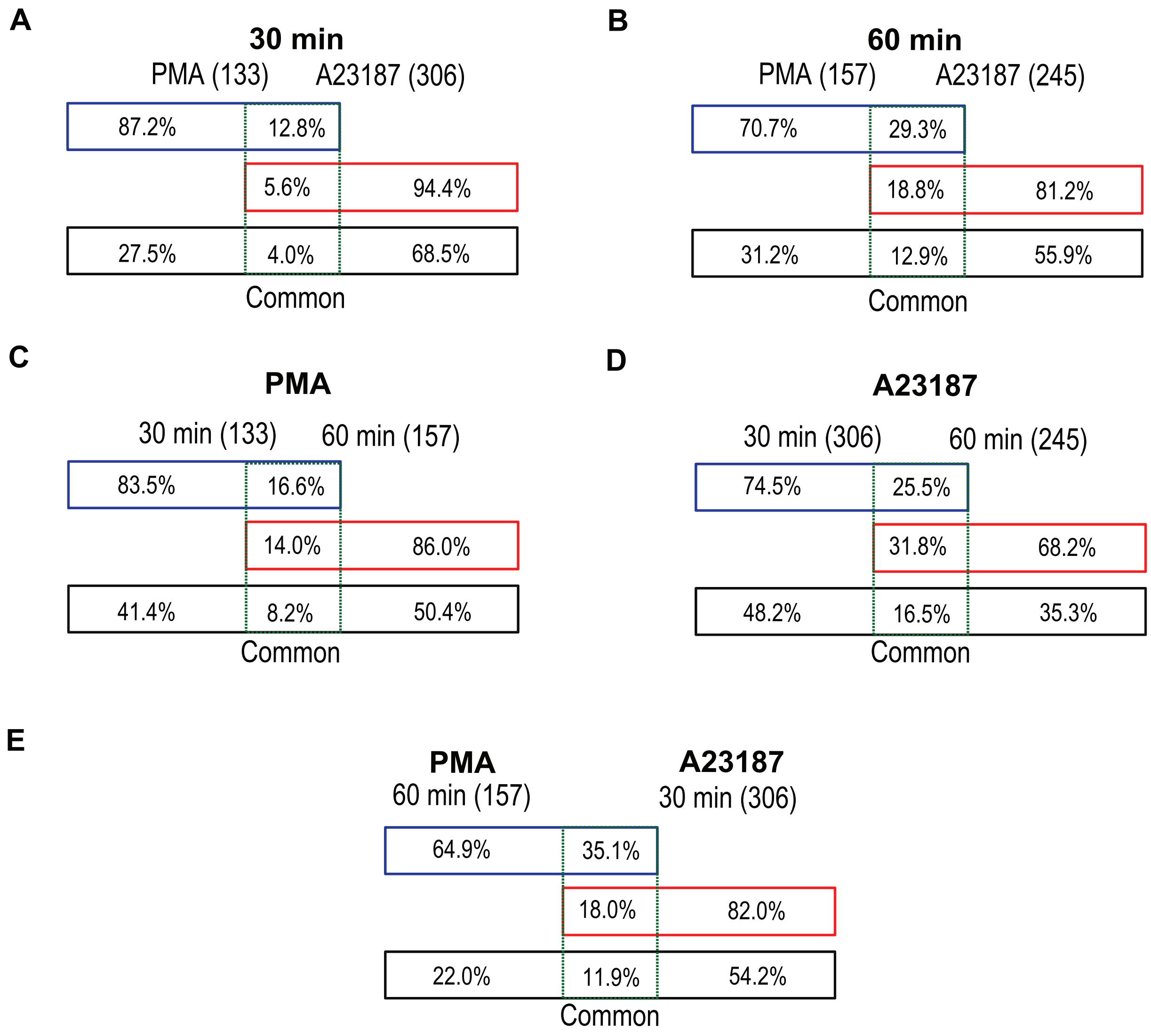

Figure S4. Percentage overlap in differentially expressed transcripts. The percentages of common and unique transcripts of coding genes showing ≥ 1.5 fold expression with $p < 0.05$ have been calculated based on each group, along with the total in both groups (shown in Figure 3).

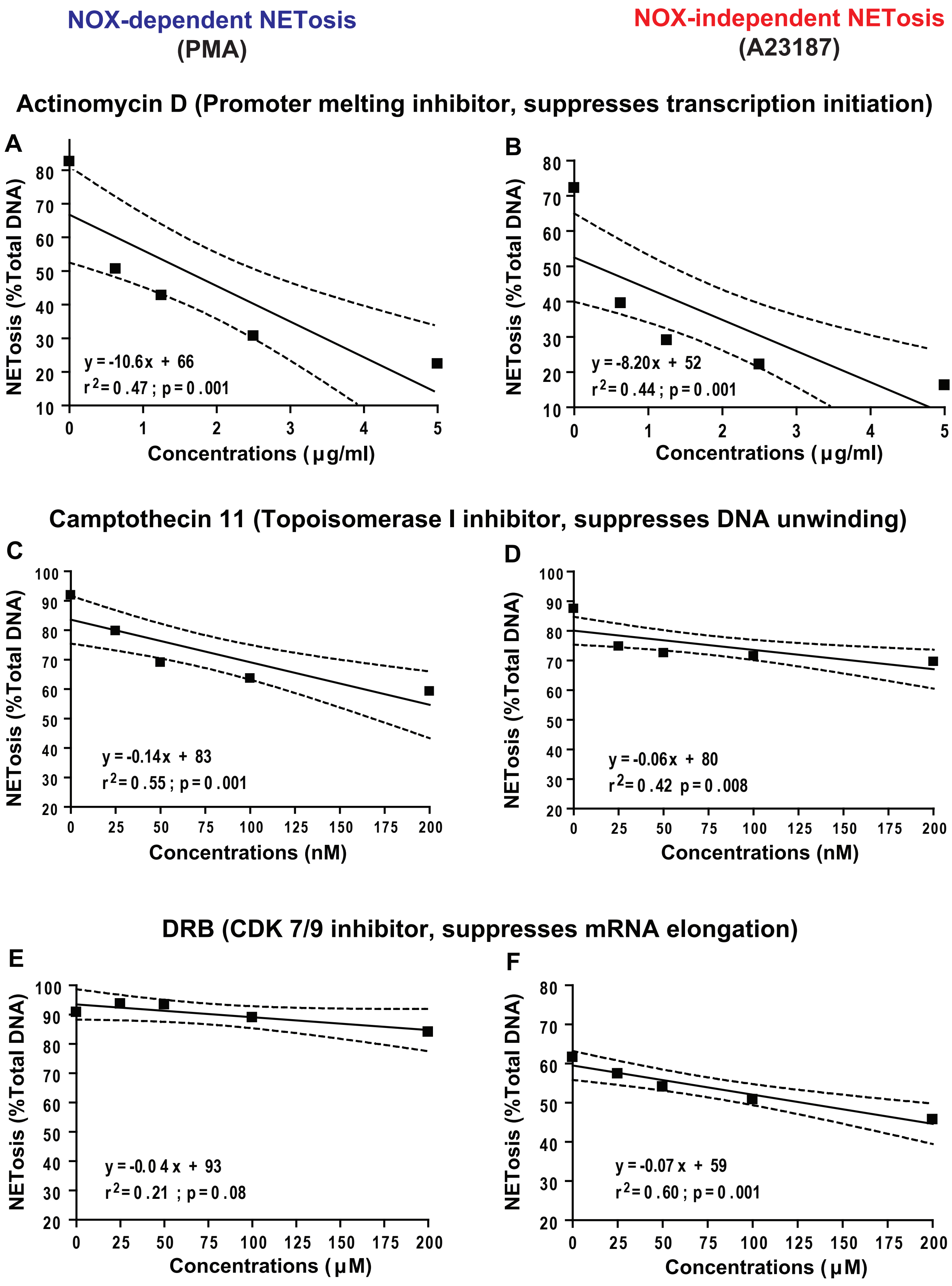

Figure S5. Sytox green assays show that inhibition of initial steps of transcription suppresses both types of NETosis. (A) Effect of G-C rich promoter melting inhibitor Actinomycin D (0, 0.625, 1.25, 2.5, 5.0 μg/ml) on PMA-mediated NETosis. **(B)** As of (A) but for A23187-mediated NETosis. **(C)** Effect of DNA topoisomerase 1 inhibitor Camptothecin 11 (0, 0.625, 1.25, 2.5, 5.0 nM) on PMA-mediated NETosis. **(D)** As of (C) but for A23187-mediated NETosis. **(E)** Effect of mRNA elongation inhibitor, that prevents the phosphorylation of CDK 7 and 9 to limit RNA polymerase movement on DNA (0, 25, 50, 100, 200 μM DRB) on PMA-mediated NETosis. **(F)** As of (E) but for A23187-mediated NETosis. n=4; p-value in each graph shows whether the slope is different than 0. Regression analysis conducted at the last time point as indicated in Figure 5.

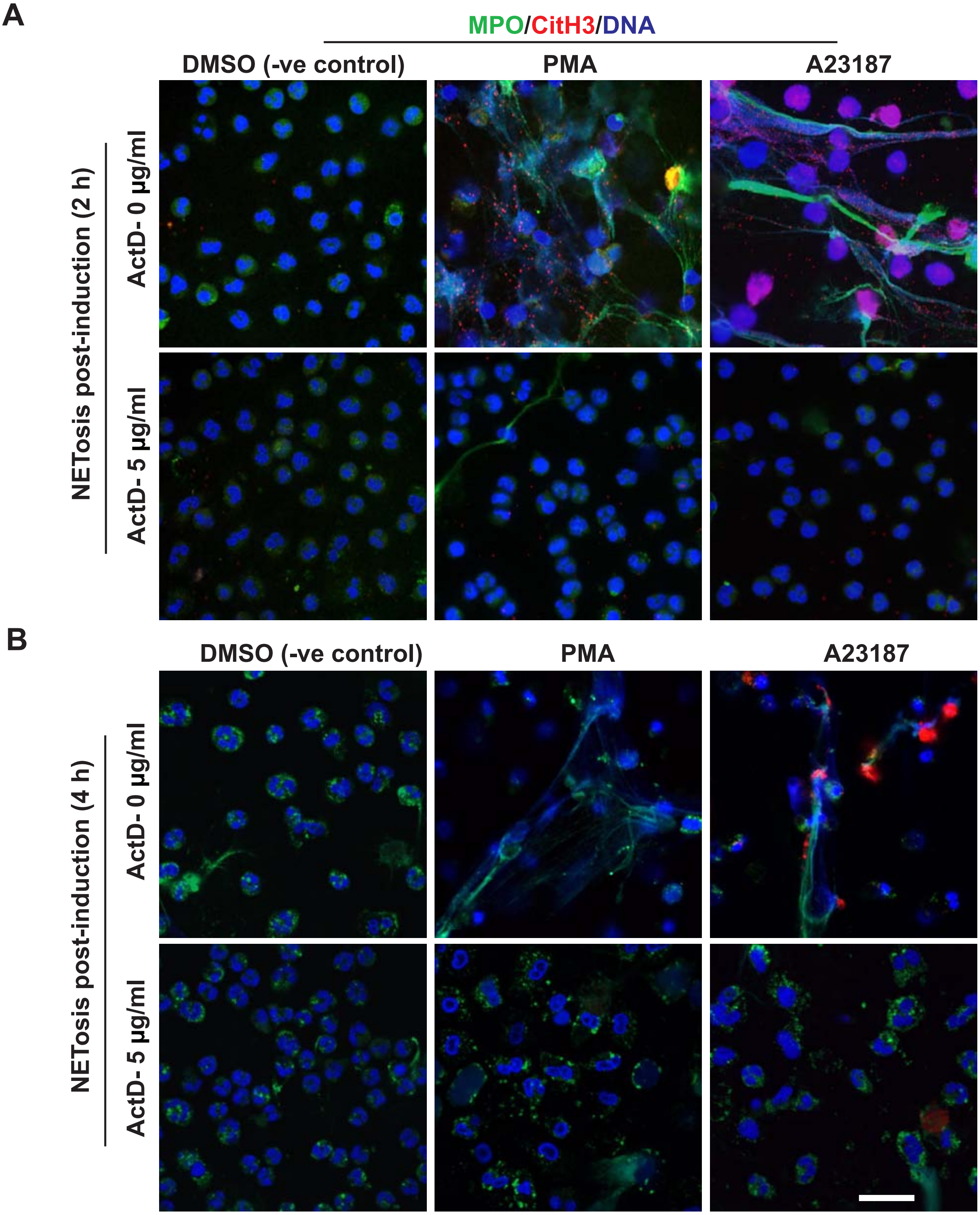

Figure S6. Confocal microscopy images show that transcription inhibitor Actinomycin D suppresses NETosis. Low magnification images. A portion of these images at 2 and 4 h are magnified to generate Figure 6. **(A)** Unstimulated control neutrophils show typical polymorphonuclear morphology. MPO is visible in the cytoplasm. MPO co-localizes to NET DNA generated by PMA-mediated NETosis. A limited amount of citrullinated histone 3 (CitH3) immunostaining is visible on some of the NETs. By contrast, intense immunostaining of CitH3 is visible on decondensed nuclei during A23187-induced NETosis and MPO colocalizes to NETs. **(B)** As of (A) but with ActD. PMA- and A23187-treated neutrophils did not show NETosis, and the nuclear morphology of these cells remains the same as that of the unstimulated control neutrophils. Only a limited amount of CitH3 is detectable in any of the ActD treated conditions. Blue, DAPI staining for DNA; Green, MPO; Red, CitH3. Scale bar, 25 µm.

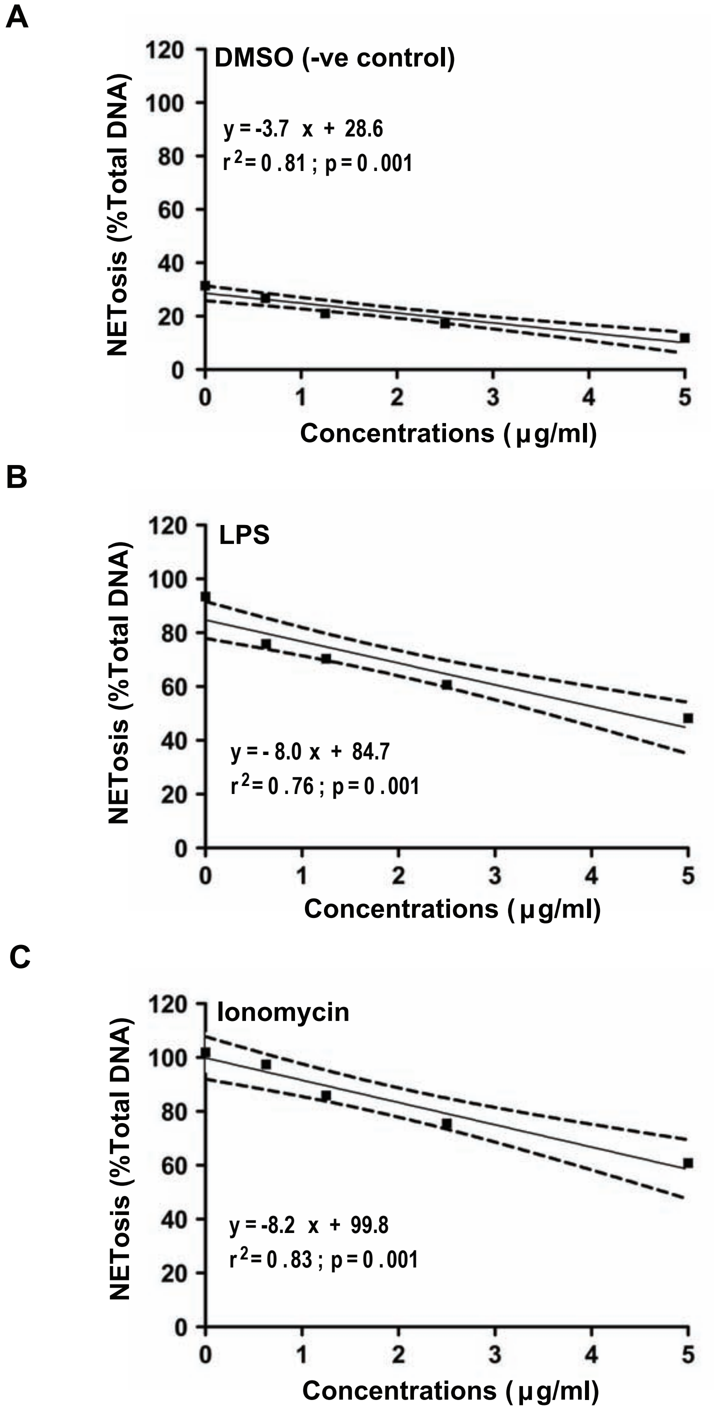

Figure S7A. Sytox green assays show that Actinomycin D suppresses both LPS-and ionomycin-mediated NETosis. Effect of G-C rich promoter melting inhibitor Actinomycin D (0, 0.625, 1.25, 2.5, 5.0 µg/ml) on (**A**) media only (DMSO, -ve control), (**B**) LPS- and (**C**) ionomycin-mediated NETosis. n=4; p-value in each graph shows whether the slope is different than 0. Regression analysis conducted at the last time point as indicated in Figure 7.

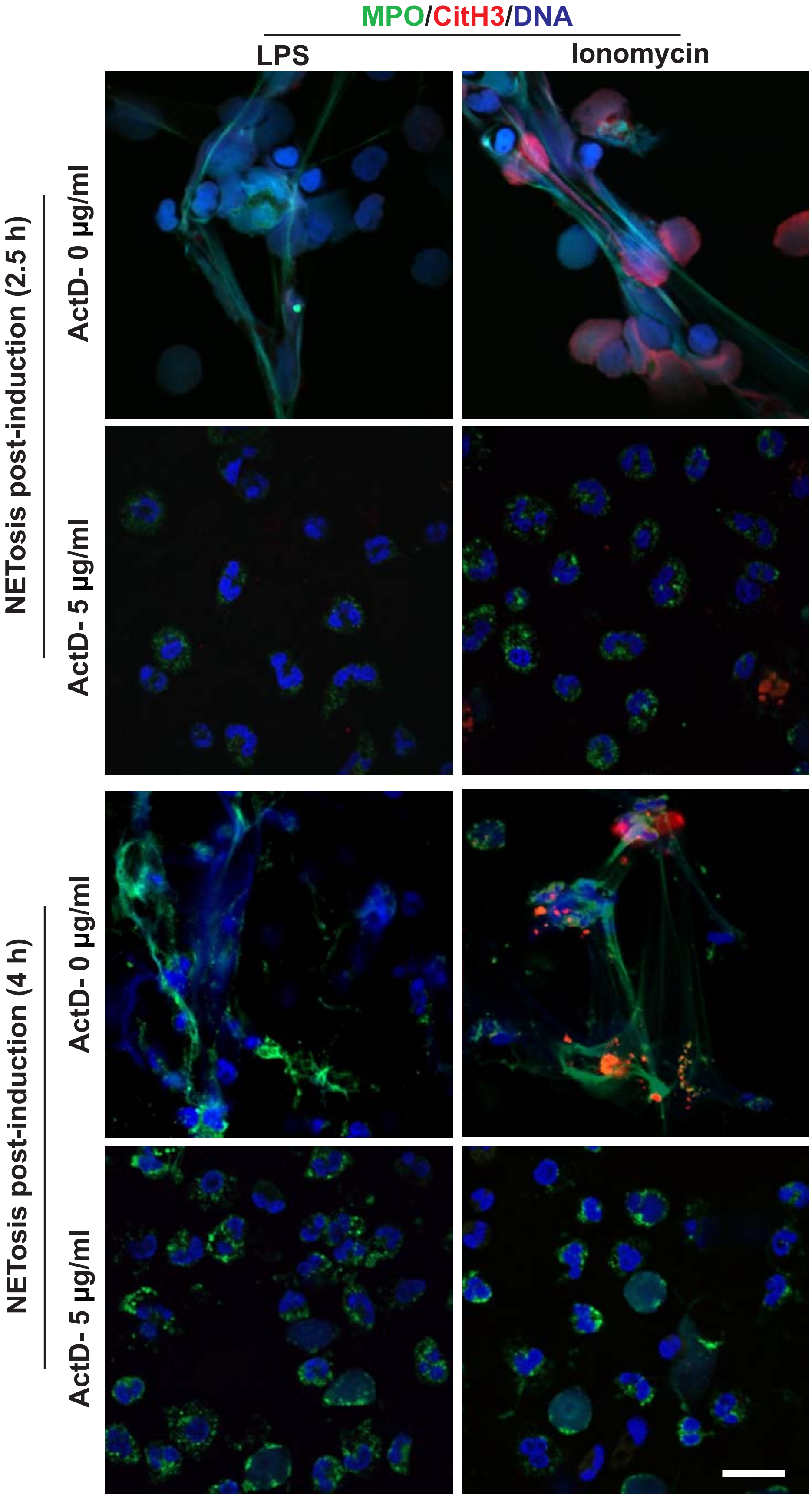

Figure S7B. Actinomycin D suppresses biologically relevant agonists LPS-and ionomycin-induced NETosis. Low magnification images. A portion of these images are magnified to genearte Figure 7C. MPO co-localizes to NET DNA generated by LPS and ionomycin-mediated NETosis. The immunostaining of CitH3 is visible on decondensed nuclei during ionomycin-induced NETosis at both time points after the NETosis induction. Blue, DAPI staining for DNA; Green, MPO; Red, CitH3. Scale bar, 25 µm.

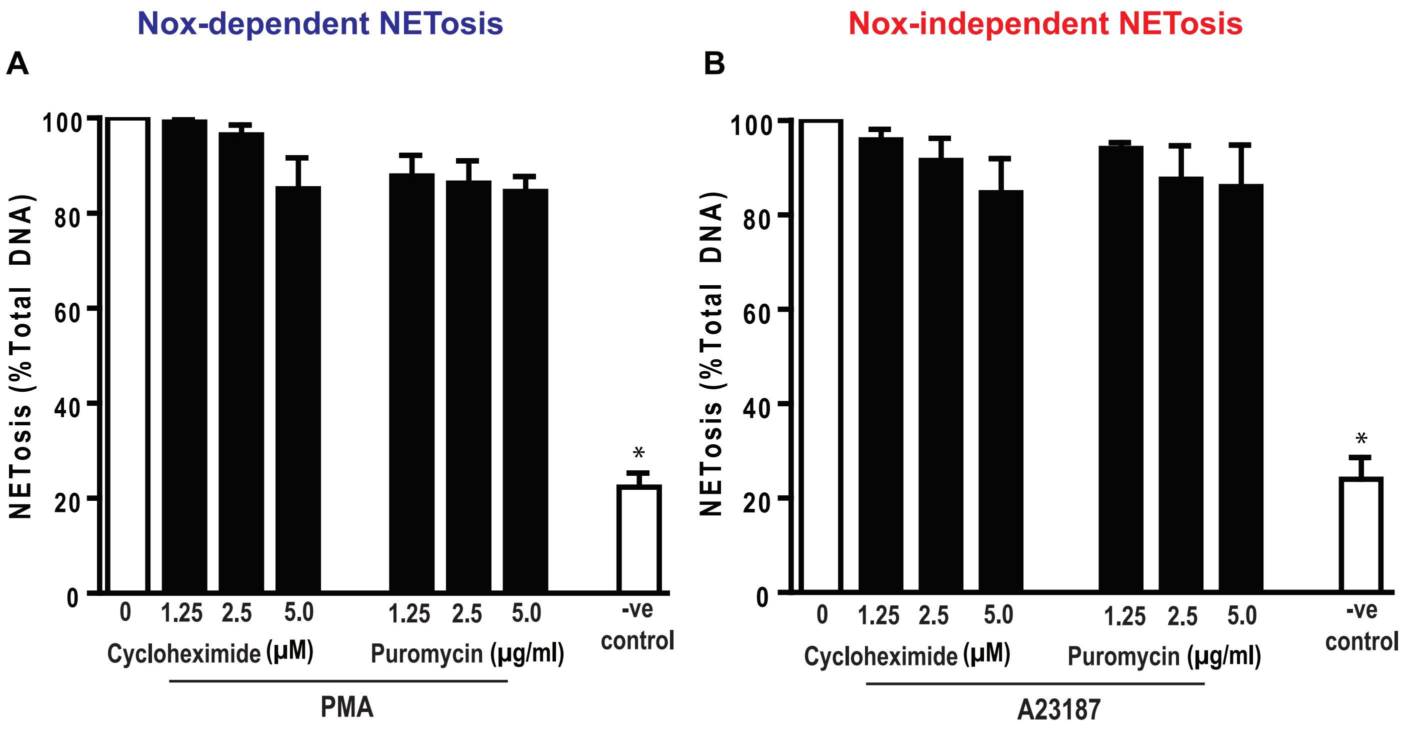

Figure S8. Translation inhibitors do not suppress both PMA-mediated Nox-dependent and A23187-mediated Nox-independent NETosis. (A) Neutrophils were preincubated with the indicated concentrations of translation inhibitor, cycloheximide or puromycin. PMA was then added to induce Nox-dependent NETosis. Degree of NETosis was monitored for 4 hours by monitoring the fluorescence intensity of Sytox green as a proxy. Sytox green dye present in the media is impermeable to living cells, and hence, when the DNA is released as NETs, Sytox green can bind and generate green florescence signal. **(B)** As of (A) except that A23187 was used, instead of PMA, for inducing Nox-independent NETosis. Percentage inhibition in each experiment was calculated, and the values were standardized to NETosis with agonists, but without any inhibitors as 100%. One sample t-test showed no significant difference between the inhibitor conditions and 100%. Only the unactivated neutrophil control is significantly different than NETotic conditions (*, $p<0.05$). For **(A)** n=4 and for **(B)** n=3.

Table S1: Primers of the target genes/transcript used in real-time PCR reactions

Gene symbol	Gene description	Refseq	Primer sequence (Forward and Reverse)
ANXA1	AnnexinA1	NM_000700	GCGAAACAATGCACAGCGTCAAC<br>CAACCTCCTCAAGGTGACCTGT
C3AR1	Complementcomponent3a receptor1	NM_004054	CCTGCTGATGTGGTCTCACCTA<br>CCTTGTGGTAGCTCAGACTCGT
CYBB	Cytochromeb-245,betapolypeptide	NM_000397	CTCTGAACTTGGAGACAGGCAAA<br>CACAGCGTGATGACAACTCCAG
EGR1	Earlygrowthresponse1	NM_001964	AGCAGCACCTTCAACCCTCAGG<br>GAGTGGTTTGGCTGGGGTAACT
GLA	Galactosidase, alpha	NM_000169	GCAACCTTGACTGCCAGGAAGA<br>CTCATAACCTGCATCCTTCCAGC
H3F3C	H3histone, family3C	NM_001013699	TCCAGAGGTTGGTGAGGGAGAT<br>TAGCGTGGATGGCACACAGGTT
IL18RAP	Interleukin18receptoraccessoryprotein	NM_003853	GCACAAAGTCCAGCGGTAACCT<br>GTCCACGAACTCACAGTATCCG
JUNB	junBproto-oncogene	NM_002229	CGATCTGCACAAGATGAACCACG<br>CTGCTGAGGTTGGTGTAAACGG
KLF2	Kruppel-likefactor2(lung)	NM_016270	CCAAGAGTTCGCATCTGAAGGC<br>CCGTGTGCTTTCGGTAGTGGC
MAP4K5	Mitogen-activated protein kinase kinase kinase kinase 5	NM_198794	CCAACTGCTGAAAGACTTCTGAC<br>CTGCTTCAGTGTAATGTGCGTGG
IL1β	Interleukin 1 beta	NM_000576	CCACAGACCTTCCAGGAGAATG<br>GTGCAGTTCAGTGATCGTACAGG
RP11-1149M10.2	Novel transcript	ENSG00000253214	TCCAGCTTGACCATTGCTCACCT<br>ATGTGGATGGTGAGCTCAGACC
SEMA7A	Semaphorin 7A	NM_001146029	CTTCTTCCGAGAGGACAATCCTG<br>GTGTTCCACTTGGAGACTGACAG
*GAPDH	GAPDH	NM_001256799	GTCTCCTCTGACTTCAACAGCG<br>ACCACCCTGTTGCTGTAGCCAA
*ACTB	Actin β	NM_001101	CACCATTGGCAATGAGCGGTTC<br>AGGTCTTTGCGGATGTCCACGT

*****housekeeping gene considered as control

Table S2: Differentially up-regulated transcripts in PMA induced NOX-dependent and A23187 induced NOX-independent NETosis.

Transcript Cluster ID	Gene Symbol	p-value	Fold expression PMA 30 min	Fold expression PMA 60 min	Fold expression A23187 30 min	Fold expression A23187 60 min
TC17002322.hg.1	AC004562.1	0.00	1.71		2.00	
TC17002062.hg.1	AC022816.2	0.02	1.53		2.79	
TC08002347.hg.1	AC069257.8	0.00	1.75			
TC10000813.hg.1	ACSL5	0.00		3.32	2.21	
TC03003258.hg.1	ACTG1	0.04	2.48			
TC10000658.hg.1	ACTG1	0.02	2.47			
TC21000785.hg.1	ADARB1	0.03		1.70	1.53	
TC11000182.hg.1	ADM	0.04			2.58	
TC22001457.hg.1	ADORA2A	0.00			1.56	
TC08000319.hg.1	AGPAT6	0.01			2.14	
TC01002479.hg.1	AK2	0.03			1.58	
TC01005394.hg.1	AK2	0.02		1.53	1.50	
TC08000937.hg.1	ALG1L13P	0.02			2.34	
TC13001557.hg.1	ALG1L13P	0.05	1.91			
TC17001807.hg.1	AMZ2P1	0.02				1.76
TC11002736.hg.1	ANKRD13D	0.03			2.19	
TC09000335.hg.1	ANXA1	0.02		2.52	2.02	
TC10002734.hg.1	ANXA11	0.04		1.94		
TC15002782.hg.1	AP3S2	0.04			2.13	
TC11001941.hg.1	AP5B1	0.00				1.54
TC05003433.hg.1	APBB3; MIR6831	0.01			1.51	
TC15000595.hg.1	ARL17A	0.05	2.35			
TC17_ctg5_hap1000002.hg.1	ARL17A	0.05			1.69	
TC02002891.hg.1	ARL4C	0.03		2.91	2.03	
TC02003640.hg.1	ARL6IP6	0.03				1.67
TC17000802.hg.1	ARSG	0.04			2.02	
TC01001777.hg.1	ATF3	0.00				1.57
TC12001216.hg.1	ATF4	0.02	2.20			
TC11000399.hg.1	ATG13	0.05			1.96	
TC11003200.hg.1	ATG2A	0.03			1.61	
TC03000766.hg.1	ATP1B3	0.01			1.84	2.02
TC01006365.hg.1	BCAS2	0.04			2.44	
TC11000544.hg.1	BEST1	0.05			1.57	
TC03000015.hg.1	BHLHE40	0.03		1.64		
TC08001043.hg.1	BIN3-IT1	0.01				1.99
TC08002251.hg.1	BIN3-IT1	0.01				1.60
TC02001696.hg.1	BRE-AS1	0.03		3.58		3.23
TC02004133.hg.1	BRE-AS1	0.04		4.67		3.60
TC11002794.hg.1	BRE-AS1	0.03	4.32			

Transcript Cluster ID	Gene Symbol	p-value	Fold expression			
			PMA		A23187	
			30 min	60 min	30 min	60 min
TC22000750.hg.1	BRE-AS1	0.04	3.83			
TC07000584.hg.1	BRI3	0.04		1.55		
TC01000567.hg.1	BTBD19	0.00				1.71
TC01001685.hg.1	BTG2	0.03			2.44	
TC04000933.hg.1	C11orf71	0.01	3.07			
TC11002312.hg.1	C11orf71	0.01		1.75	1.51	1.56
TC15000691.hg.1	C15orf39	0.04			2.07	
TC15000357.hg.1	C15orf48; MIR147B	0.01			2.10	
TC12001171.hg.1	C3AR1	0.02			6.55	
TC08001266.hg.1	CA2	0.02	2.85			
TC0X000070.hg.1	CA5BP1	0.01				1.67
TC19000496.hg.1	CAPNS1	0.03			1.81	
TC11000327.hg.1	CAT	0.04		1.70	1.70	
TC17000383.hg.1	CCL2	0.00			7.27	4.55
TC17001385.hg.1	CCL3	0.00			7.21	4.95
TC17001394.hg.1	CCL3	0.00			6.25	4.15
TC17002594.hg.1	CCL3	0.00			5.53	4.48
TC17001388.hg.1	CCL3L3; CCL3L1	0.00			6.57	4.27
TC17001395.hg.1	CCL3L3; CCL3L1	0.00			7.77	4.89
TC17000408.hg.1	CCL4; LOC101060278	0.01			4.36	1.94
TC17000416.hg.1	CCL4L1; CCL4L2	0.00			4.21	2.54
TC17000423.hg.1	CCL4L1; CCL4L2	0.00			4.37	2.82
TC17001378.hg.1	CCL5	0.01				1.84
TC03000257.hg.1	CCRL2	0.03			2.51	
TC05001861.hg.1	CD14	0.02			2.32	
TC01005001.hg.1	CD24	0.04	1.59			
TC0Y000182.hg.1	CD24	0.03				1.84
TC19000729.hg.1	CD37	0.04			1.62	
TC0X000666.hg.1	CD40LG	0.02			1.59	
TC01005732.hg.1	CD58	0.05			4.23	
TC09001085.hg.1	CD69	0.04	6.03			
TC12001207.hg.1	CD69	0.04		3.23	2.24	9.65
TC12002752.hg.1	CD69	0.03		3.48	2.22	8.80
TC22001130.hg.1	CD69	0.04	5.87			
TC06000099.hg.1	CD83	0.00				2.15
TC01000281.hg.1	CDC42-IT1	0.02			1.62	
TC19002491.hg.1	CEACAM1	0.03		1.82		
TC18000059.hg.1	CHMP1B	0.01				2.22
TC14001732.hg.1	CHURC1	0.04			2.48	
TC03001156.hg.1	CIDECP	0.03			1.97	
TC09000415.hg.1	CKS2	0.01		1.77		1.80

Transcript Cluster ID	Gene Symbol	p-value	Fold expression			
			PMA		A23187	
			30 min	60 min	30 min	60 min
TC09002074.hg.1	CKS2	0.02		2.45		2.42
TC02004719.hg.1	COMMD5	0.04	2.75			
TC08002624.hg.1	COMMD5; LOC101928879	0.01			1.53	
TC04001346.hg.1	COQ2	0.05			2.85	
TC03001095.hg.1	COX17	0.04	1.91			
TC10000238.hg.1	CREM	0.02			2.35	
TC01000955.hg.1	CSF1	0.00				2.53
TC03001302.hg.1	CSRNP1	0.02				2.34
TC20000428.hg.1	CSTF1	0.01			1.95	
TC0X001836.hg.1	CTB-11I22.2	0.03	1.61			
TC04000411.hg.1	CXCL1	0.04		3.49		
TC17001029.hg.1	CXCL16	0.04			3.56	
TC04001286.hg.1	CXCL2	0.02				2.05
TC0X000711.hg.1	CXorf40A	0.01			1.53	
TC0X001909.hg.1	CXorf40A	0.04			1.61	1.56
TC12000527.hg.1	CYB5D1	0.00	3.34			
TC17000119.hg.1	CYB5D1	0.02		2.81		1.64
TC0X000171.hg.1	CYBB	0.02			3.33	
TC02001311.hg.1	CYP27A1	0.01			1.52	
TC22000666.hg.1	DDIT3	0.03	6.67			
TC0Y000053.hg.1	DDX3Y	0.04				2.32
TC17001058.hg.1	DERL2	0.03			1.64	
TC22000829.hg.1	DESI1	0.03			1.53	
TC08001044.hg.1	DKFZp434E1119	0.00	6.28			
TC11002794.hg.1	DKFZp434E1119	0.00		3.97	2.56	4.19
TC11000647.hg.1	DRAP1	0.02				1.64
TC05002066.hg.1	DUSP1	0.01			2.72	3.14
TC01003847.hg.1	DUSP10	0.03				1.50
TC02002102.hg.1	DUSP2	0.01				1.51
TC14000471.hg.1	DUSP2	0.04	1.82			
TC0X000952.hg.1	DYNLT3	0.03			1.59	
TC01002322.hg.1	ECE1	0.04			2.81	
TC01005311.hg.1	ECE1	0.03			2.76	
TC04002396.hg.1	EGR1	0.00	23.79			
TC05000701.hg.1	EGR1	0.01		6.59	5.05	2.78
TC10001327.hg.1	EGR2	0.00			2.58	2.03
TC22000648.hg.1	EGR2	0.04	1.69			
TC08001044.hg.1	EGR3	0.00			4.29	3.19
TC08002253.hg.1	EGR3	0.00		3.80	8.58	6.78
TC19002413.hg.1	EGR3	0.01	4.30			
TC20000283.hg.1	EGR3	0.00	2.09			

Transcript Cluster ID	Gene Symbol	p-value	Fold expression			
			PMA		A23187	
			30 min	60 min	30 min	60 min
TC11001915.hg.1	EHD1	0.04			2.00	
TC17000119.hg.1	EIF1	0.04	3.21			
TC17001933.hg.1	EIF4A3	0.04			2.28	
TC0X000768.hg.1	EMD	0.01			1.52	
TC12000189.hg.1	EMP1	0.01				2.03
TC04000414.hg.1	EREG	0.02				2.61
TC17000965.hg.1	FAM101B	0.03			3.26	
TC06003239.hg.1	FAM120B	0.04			1.50	
TC0X000777.hg.1	FAM223A; FAM223B	0.01			1.50	
TC0X001540.hg.1	FAM223A; FAM223B	0.04			1.57	
TC05000698.hg.1	FAM53C	0.04			1.65	
TC10000523.hg.1	FAM85A	0.04	1.70			
TC19000885.hg.1	FCAR	0.02			3.29	
TC19002237.hg.1	FCAR	0.02			3.44	
TC19000470.hg.1	FFAR2	0.03				2.67
TC19000467.hg.1	FFAR3	0.00				1.72
TC01002530.hg.1	FHL3	0.04			1.51	1.52
TC13000354.hg.1	FKSG29	0.03				2.12
TC13001253.hg.1	FKSG29	0.01				2.00
TC12002387.hg.1	FKSG49	0.02	1.64			
TC15001352.hg.1	FLJ10038	0.02				1.92
TC15002531.hg.1	FLJ10038	0.03				1.82
TC02000950.hg.1	FLJ46875	0.03	2.00			
TC02002446.hg.1	FLJ46875	0.00			4.40	2.86
TC02004656.hg.1	FLJ46875	0.01			2.67	1.93
TC02004656.hg.1	FLJ46875	0.00				
TC12002088.hg.1	FLJ46875	0.03	2.48			
TC0X001530.hg.1	FLNA	0.02		1.82	2.17	
TC14000471.hg.1	FOS	0.00		4.00	6.28	5.70
TC16001990.hg.1	FOS	0.00	5.55			
TC12000953.hg.1	FOSB	0.02	2.84			
TC12002399.hg.1	FOSB	0.02	3.25			
TC19000647.hg.1	FOSB	0.00		3.34	4.69	9.26
TC19002172.hg.1	FOSB	0.00		3.14	3.64	7.93
TC02000183.hg.1	FOSL2	0.03		2.63	2.05	
TC19001787.hg.1	FPR1	0.03			1.92	
TC19002215.hg.1	FPR1	0.04			1.97	
TC11001860.hg.1	FTH1	0.03			1.50	
TC11003179.hg.1	FTH1	0.01			1.67	
TC02004131.hg.1	FTH1P3	0.01		1.53	1.51	
TC0X001156.hg.1	FTX	0.03			1.71	

Transcript Cluster ID	Gene Symbol	p-value	Fold expression			
			PMA		A23187	
			30 min	60 min	30 min	60 min
TC03000349.hg.1	G0S2	0.04	2.90			
TC08001246.hg.1	G0S2	0.04	2.85			
TC17000915.hg.1	GAA	0.02				1.51
TC07000198.hg.1	GARS	0.05		3.89		
TC14001152.hg.1	GCH1	0.04			2.36	
TC14002019.hg.1	GCH1	0.03			2.63	
TC03003122.hg.1	GK5	0.01		1.52		
TC0X000142.hg.1	GK-IT1	0.04			1.72	
TC0X001219.hg.1	GLA	0.04			2.52	
TC14001791.hg.1	GLA	0.04	4.44			
TC17001031.hg.1	GLTPD2	0.01			2.33	
TC05001879.hg.1	GNPDA1	0.04		1.52		
TC19000442.hg.1	GPI	0.04				1.57
TC01000809.hg.1	GPR174	0.04	1.72			
TC13000828.hg.1	GPR18	0.04		1.87		
TC13000829.hg.1	GPR183	0.04				2.71
TC03001407.hg.1	GPX1	0.00				1.84
TC17000570.hg.1	GRN	0.03			1.87	
TC22000304.hg.1	GTPBP1	0.02			1.68	
TC12001368.hg.1	H3F3C	0.01			1.85	
TC01001269.hg.1	HAX1	0.01			2.16	1.56
TC16000008.hg.1	HBA1; HBA2	0.03			1.59	
TC05001854.hg.1	HBEGF	0.00				2.05
TC12000141.hg.1	HCAR2	0.04	4.66			
TC12002078.hg.1	HCAR2	0.01		5.06		4.92
TC06000299.hg.1	HCG14	0.04			1.73	
TC6_apd_hap1000001.hg.1	HCG14	0.04			1.73	
TC6_cox_hap2000003.hg.1	HCG14	0.03			1.80	
TC6_mcf_hap5000001.hg.1	HCG14	0.03			1.80	
TC6_qbl_hap6000001.hg.1	HCG14	0.04			1.73	
TC6_ssto_hap7000001.hg.1	HCG14	0.03			1.80	
TC05002152.hg.1	HEIH	0.04		1.78		
TC03001071.hg.1	HES1	0.03		2.30		
TC03002732.hg.1	HES1	0.02		2.94	1.67	
TC17000231.hg.1	HES1	0.02	2.51			
TC17001237.hg.1	HES1	0.02	3.76			
TC08002304.hg.1	HEXA-AS1	0.03	2.05			
TC11000750.hg.1	HEXA-AS1	0.03	1.93			
TC15000657.hg.1	HEXA-AS1	0.02		2.03		

Transcript Cluster ID	Gene Symbol	p-value	Fold expression			
			PMA		A23187	
			30 min	60 min	30 min	60 min
TC15002266.hg.1	HEXA-AS1	0.02		2.21		
TC17000019.hg.1	HIC1	0.04		1.51		
TC07001534.hg.1	HIP1	0.04				1.50
TC06000171.hg.1	HIST1H2BF; HIST1H2BG; HIST1H2BE; HIST1H2BI; HIST1H2BC	0.02			1.60	
TC06000166.hg.1	HIST1H4C; HIST1H4I; HIST1H4A; HIST1H4D; HIST1H4F; HIST1H4K; HIST1H4J; HIST1H4H; HIST1H4B; HIST1H4E; HIST1H4L; HIST2H4A; HIST4H4; HIST2H4B	0.01				1.60
TC06001424.hg.1	HIST1H4K; HIST1H4I; HIST1H4A; HIST1H4D; HIST1H4F; HIST1H4J; HIST1H4C; HIST1H4H; HIST1H4B; HIST1H4E; HIST1H4L; HIST2H4A; HIST4H4; HIST2H4B	0.03			1.54	
TC01001174.hg.1	HIST2H2AA4; HIST2H2AA3	0.02			6.20	
TC01003190.hg.1	HIST2H2AA4; HIST2H2AA3	0.01			2.97	1.71
TC01006326.hg.1	HIST2H2AC	0.04			2.19	1.59
TC01001046.hg.1	HIST2H2BA	0.02			1.71	
TC01003191.hg.1	HIST2H2BC	0.01			2.24	
TC01003193.hg.1	HIST2H2BE	0.01			3.63	1.57
TC01003172.hg.1	HIST2H2BF; HIST2H3PS2	0.00			3.25	
TC01003187.hg.1	HIST2H2BF; RP11-196G18.3; RP11-196G18.21	0.00			1.92	
TC01001173.hg.1	HIST2H4B; HIST2H4A; HIST1H4I; HIST1H4A;	0.04			1.95	

Transcript Cluster ID	Gene Symbol	p-value	Fold expression PMA 30 min	Fold expression PMA 60 min	Fold expression A23187 30 min	Fold expression A23187 60 min
TC01003192.hg.1	HIST1H4D; HIST1H4F; HIST1H4K; HIST1H4J; HIST1H4C; HIST1H4H; HIST1H4B; HIST1H4E; HIST1H4L; HIST4H4 HIST2H4B; HIST2H4A; HIST1H4I; HIST1H4A; HIST1H4D; HIST1H4F; HIST1H4K; HIST1H4J; HIST1H4C; HIST1H4H; HIST1H4B; HIST1H4E; HIST1H4L; HIST4H4	0.04			1.94	
TC06002084.hg.1	HNRNPA3P3	0.04	1.55			
TC17000165.hg.1	HS3ST3B1	0.01			1.79	
TC17001232.hg.1	HS3ST3B1	0.01	1.54			
TC09001589.hg.1	HSPA5	0.04			2.87	
TC01005882.hg.1	HSPC081	0.03				1.67
TC19000175.hg.1	ICAM4	0.01				2.12
TC19000237.hg.1	IER2	0.00				1.55
TC06001510.hg.1	IER3	0.02		2.47		
TC6_cox_hap2000143.hg.1	IER3	0.04		2.42		
TC6_dbb_hap3000132.hg.1	IER3	0.04		2.42		
TC6_mann_hap4000121.hg.1	IER3	0.04		2.42		
TC6_qbl_hap6000133.hg.1	IER3	0.04		2.31		
TC6_ssto_hap7000123.hg.1	IER3	0.04		2.42		
TC01001569.hg.1	IER5	0.01				1.50
TC11002312.hg.1	IFI30	0.04	2.39			
TC19002629.hg.1	IFI30	0.04		1.83		
TC10000637.hg.1	IFIT3	0.02		2.05		
TC12001696.hg.1	IFNG	0.01				1.62
TC12002948.hg.1	IFNG	0.04			1.51	1.80
TC21000408.hg.1	IL10RB-AS1	0.02				1.57
TC21000932.hg.1	IL10RB-AS1	0.02				1.64

Transcript Cluster ID	Gene Symbol	p-value	Fold expression			
			PMA		A23187	
			30 min	60 min	30 min	60 min
TC11002771.hg.1	IL18BP	0.03				1.66
TC02000623.hg.1	IL18RAP	0.05			3.92	
TC02002218.hg.1	IL1A	0.02				2.17
TC02002219.hg.1	IL1B	0.01			1.59	1.73
TC03001048.hg.1	IL1RAP	0.05			6.72	2.89
TC02004994.hg.1	IL1RL1	0.05				2.08
TC02000720.hg.1	IL1RN	0.01				1.88
TC0X000008.hg.1	IL3RA	0.04		2.25		
TC03001122.hg.1	IL5RA	0.03				2.25
TC07001318.hg.1	INHBA	0.01				1.74
TC11001241.hg.1	IRF7	0.04				1.64
TC01003351.hg.1	ISG20L2	0.02			2.70	1.77
TC01003901.hg.1	ITPKB	0.01				1.52
TC01006164.hg.1	ITPKB	0.02			3.33	3.41
TC01001864.hg.1	ITPKB-AS1	0.02			2.01	1.60
TC06001288.hg.1	JARID2-AS1	0.01			2.39	1.65
TC06003492.hg.1	JARID2-AS1	0.01			2.08	1.77
TC22000796.hg.1	JOSD1	0.03		2.32	1.65	
TC01002708.hg.1	JUN	0.00				1.98
TC19001995.hg.1	JUNB	0.04				2.27
TC19001285.hg.1	JUND	0.04			2.85	2.74
TC08002345.hg.1	KATNBL1	0.01	6.47			
TC15001191.hg.1	KATNBL1	0.01		4.55		
TC08000640.hg.1	KB-1507C5.4	0.03				2.49
TC17000165.hg.1	KB-1507C5.4	0.02	2.28			
TC03000407.hg.1	KBTBD8	0.02				1.68
TC19000321.hg.1	KLF2	0.00			3.10	4.08
TC09001447.hg.1	KLF4	0.00				1.50
TC17000311.hg.1	KSR1	0.04				1.51
TC22000647.hg.1	LIF	0.00				2.04
TC19002719.hg.1	LILRA5	0.02			1.69	
TC02000667.hg.1	LIMS1; LIMS3L	0.01		1.98		
TC02004997.hg.1	LIMS3L; LIMS3	0.00		1.56		
TC17001233.hg.1	LINC00664	0.01	4.00			
TC19000392.hg.1	LINC00664	0.00		2.63	2.13	2.04
TC19002042.hg.1	LINC00664	0.00	3.22	3.33	2.67	2.68
TC05001066.hg.1	LINC00847	0.00		1.51		
TC07002289.hg.1	LINC00847	0.04	2.53			
TC17000118.hg.1	LINC00847	0.04	2.20			
TC03001551.hg.1	LINC00877	0.05			1.91	
TC03002966.hg.1	LINC00877	0.05			2.08	
TC03001074.hg.1	LINC00884	0.04		1.84		

Transcript Cluster ID	Gene Symbol	p-value	Fold expression			
			PMA		A23187	
			30 min	60 min	30 min	60 min
TC03002737.hg.1	LINC00884	0.01		1.94	1.51	
TC03002738.hg.1	LINC00884	0.02		1.92		
TC19001354.hg.1	LINC00884	0.03	1.64			
TC16001394.hg.1	LINC00921	0.04			2.34	
TC0X002289.hg.1	LINC00936	0.05	3.13			
TC12000698.hg.1	LINC00936	0.00		5.32	3.93	4.06
TC01006046.hg.1	LINC01136	0.02		1.91		1.66
TC01006046.hg.1	LINC01136	0.01				
TC08000517.hg.1	LINC01136	0.03	1.68			
TC01003720.hg.1	LINC01136; RP11-134P9.1	0.01		2.23	1.85	1.77
TC14000472.hg.1	LINC01220	0.00		2.04	2.64	2.48
TC14001775.hg.1	LINC01220	0.00		1.94	2.46	2.61
TC08001414.hg.1	LINC01262	0.00	4.74			
TC04000933.hg.1	LINC01262; RP11-462G22.1	0.02		2.87		2.03
TC20000401.hg.1	LINC01272; RP11-290F20.3	0.04			1.67	
TC04001178.hg.1	LINC01366	0.04	3.33			
TC14000500.hg.1	LINC01366	0.02	2.75			
TC05000934.hg.1	LINC01366; CTB-114C7.3	0.01				1.78
TC10000629.hg.1	LIPN	0.01			8.21	
TC12002167.hg.1	LOC100049716	0.03			1.61	
TC05002051.hg.1	LOC100128059; CTB-114C7.4	0.02		2.69		
TC06004142.hg.1	LOC100129518; SOD2	0.00				1.56
TC06003475.hg.1	LOC100130357	0.03		2.12		
TC01003855.hg.1	LOC100132062	0.05			1.75	
TC16000936.hg.1	LOC100190986	0.01			1.55	
TC10000965.hg.1	LOC100288619	0.02				1.53
TC01005823.hg.1	LOC100507670	0.02		1.76		1.71
TC01003222.hg.1	LOC100507670; RP11-126K1.2	0.01				1.51
TC01003401.hg.1	LOC101927018	0.01	1.84			
TC04001822.hg.1	LOC101927018	0.02	1.71			
TC17000335.hg.1	LOC101927018	0.04		1.64		
TC17002147.hg.1	LOC101927018	0.03		1.56	1.69	
TC10000784.hg.1	LOC101927472; RP11-127L20.3	0.03			1.50	1.96
TC14001195.hg.1	LOC101927780	0.02		1.54		
TC07000677.hg.1	LOC101927902; RP11-325F22.2	0.01			1.72	
TC09001436.hg.1	LOC101928579; RP11-217B7.3	0.00		1.99		1.60
TC11002137.hg.1	LOC101928865	0.02		1.99		

Transcript Cluster ID	Gene Symbol	p-value	Fold expression			
			PMA		A23187	
			30 min	60 min	30 min	60 min
TC11003263.hg.1	LOC101928865	0.01		2.20	1.72	1.59
TC20000598.hg.1	LOC101929207; RP5-1022P6.4	0.00			1.78	
TC12002736.hg.1	LOC101929610	0.00	2.14			
TC13000182.hg.1	LOC284454	0.02	3.22			
TC19002349.hg.1	LOC284454	0.00		1.82	2.84	2.96
TC01004218.hg.1	LOC284513	0.00				1.58
TC08002252.hg.1	LOC286058	0.00		2.55		4.09
TC11000545.hg.1	LOC399900	0.03		2.23	1.94	2.04
TC14001010.hg.1	LOC647859	0.03	1.54			
TC08002253.hg.1	LOC728445	0.04	4.56			
TC08002305.hg.1	LOC728445	0.01		3.88	3.83	3.20
TC04002882.hg.1	LOC731424	0.00				1.63
TC04001811.hg.1	LOC731424; RP11-701P16.5	0.01				1.81
TC06002446.hg.1	LST1	0.03			2.46	
TC06003347.hg.1	LTB	0.03			2.90	
TC01003998.hg.1	LYST-AS1	0.04			2.82	
TC12000611.hg.1	LYZ	0.01			2.03	
TC22000297.hg.1	MAFF	0.00				1.56
TC14001120.hg.1	MAP4K5	0.03		1.61		
TC19000128.hg.1	MCOLN1	0.03			2.29	
TC0X001525.hg.1	MECP2	0.04			1.65	
TC17000959.hg.1	METRNL	0.02				1.67
TC0X000111.hg.1	MGC12916	0.03	1.97			
TC17000166.hg.1	MGC12916	0.03			5.33	
TC0X000180.hg.1	MID1IP1	0.05			1.95	
TC0X000985.hg.1	MIR221	0.01		3.39		3.05
TC0X000986.hg.1	MIR222	0.00		3.09		2.72
TC0X000356.hg.1	MIR223	0.05			2.99	
TC17000983.hg.1	MIR22HG; MIR22	0.04				1.52
TC07000773.hg.1	MIR23A	0.02	2.48			
TC19001224.hg.1	MIR23A; MIR24-2; MIR27A; LOC284454	0.00			2.59	2.95
TC09000416.hg.1	MIR3153	0.02				1.74
TC09002503.hg.1	MIR3153	0.03	2.46			
TC0X000007.hg.1	MIR3690	0.01			2.98	
TC04001812.hg.1	MIR3945	0.01			2.04	2.01
TC0X001529.hg.1	MIR4308	0.03	1.54			
TC02000369.hg.1	MIR4434	0.00				1.65
TC17001883.hg.1	MIR4738	0.00			10.54	11.98
TC04001138.hg.1	MIR4802	0.02				1.80
TC04000563.hg.1	MIR576	0.02			1.76	

Transcript Cluster ID	Gene Symbol	p-value	Fold expression PMA 30 min	PMA 60 min	A23187 30 min	A23187 60 min
TC02000266.hg.1	MIR616	0.03	15.65			
TC13000355.hg.1	MIR623	0.03				1.60
TC20000363.hg.1	MMP9	0.04			2.34	
TC17002717.hg.1	MTVR2	0.04				1.63
TC21000189.hg.1	MX1	0.00		1.81		
TC21000739.hg.1	MX1	0.00		3.43		
TC03000187.hg.1	MYD88	0.04			2.08	
TC05000831.hg.1	NDST1	0.01			1.63	
TC12000113.hg.1	NECAP1	0.05			2.96	
TC06001552.hg.1	NELFE; MIR1236	0.00			1.56	
TC6_cox_hap2000180.hg.1	NELFE; MIR1236	0.00			1.50	
TC6_dbb_hap3000169.hg.1	NELFE; MIR1236	0.00			1.50	
TC6_mcf_hap5000155.hg.1	NELFE; MIR1236	0.00			1.50	
TC6_qbl_hap6000170.hg.1	NELFE; MIR1236	0.00			1.50	
TC6_ssto_hap7000148.hg.1	NELFE; MIR1236	0.00			1.50	
TC06001548.hg.1	NEU1	0.00				2.45
TC6_apd_hap1000089.hg.1	NEU1	0.00				2.45
TC6_cox_hap2000175.hg.1	NEU1	0.00				2.43
TC6_dbb_hap3000164.hg.1	NEU1	0.00				2.45
TC6_mann_hap4000146.hg.1	NEU1	0.00				2.45
TC6_mcf_hap5000150.hg.1	NEU1	0.00				2.45
TC6_qbl_hap6000165.hg.1	NEU1	0.00				2.45
TC6_ssto_hap7000144.hg.1	NEU1	0.00				2.45
TC09001325.hg.1	NFIL3	0.02			4.31	2.86
TC19001457.hg.1	NFKBID	0.02				1.50
TC0X000393.hg.1	NHSL2	0.04			2.62	
TC16000442.hg.1	NOD2	0.03			1.76	
TC16001549.hg.1	NOD2	0.02			1.99	
TC16000180.hg.1	NOMO1	0.05			1.83	
TC16000912.hg.1	NOMO2	0.04			1.86	
TC16000194.hg.1	NOMO3; NOMO2	0.05			1.89	
TC17001954.hg.1	NPLOC4	0.03			1.81	
TC06004062.hg.1	NQO2	0.03			1.83	
TC01003765.hg.1	NR4A1	0.02	1.66			
TC12000414.hg.1	NR4A1	0.00		1.81	2.01	1.98

Transcript Cluster ID	Gene Symbol	p-value	Fold expression			
			PMA		A23187	
			30 min	60 min	30 min	60 min
TC02002445.hg.1	NR4A2	0.01			3.55	3.78
TC01000226.hg.1	NR4A3	0.02	6.37			
TC08001246.hg.1	NSMAF	0.00		1.83		
TC15000547.hg.1	NSMAF	0.01	1.98			
TC12002572.hg.1	OAS1	0.03				2.00
TC12000414.hg.1	OCLM	0.03	1.94			
TC03001071.hg.1	OLR1	0.00	2.23			
TC12001216.hg.1	OLR1	0.00		5.87	5.80	6.18
TC14000873.hg.1	OR4K14	0.04				1.78
TC09002907.hg.1	ORM1	0.05		1.72		1.57
TC09002906.hg.1	ORM2	0.03		1.71		
TC17001331.hg.1	OSM	0.00	6.87			
TC22000648.hg.1	OSM	0.00		5.06	4.18	3.80
TC14000131.hg.1	OXA1L	0.05			1.64	
TC0X000427.hg.1	P2RY10	0.00		2.06		
TC03001904.hg.1	P2RY14	0.01		1.59		
TC01000232.hg.1	PADI4	0.01			2.11	
TC21000401.hg.1	PAXBP1	0.04				1.64
TC21000401.hg.1	PAXBP1	0.05		2.21		
TC21000925.hg.1	PAXBP1	0.04		2.84		
TC07001618.hg.1	PDK4	0.05				2.11
TC19001640.hg.1	PGLYRP1	0.00			1.59	
TC0X001993.hg.1	PIGA	0.02				1.50
TC04001376.hg.1	PIGY; PYURF	0.02				1.50
TC10000475.hg.1	PLAU	0.04		3.90		
TC19000545.hg.1	PLEKHG2	0.02				1.58
TC17000226.hg.1	PLIN2	0.02	11.81			
TC17001231.hg.1	PLIN2	0.03	7.83			
TC01000566.hg.1	PLK3	0.04				1.75
TC0X000267.hg.1	PLP2	0.04			1.87	
TC12002519.hg.1	PLXNC1	0.05			2.43	
TC18000213.hg.1	PMAIP1	0.04			3.83	2.65
TC18000741.hg.1	PMAIP1	0.05		3.57		
TC07001666.hg.1	PMS2P1	0.02			1.61	
TC07003082.hg.1	PMS2P1	0.02			1.70	
TC14000056.hg.1	PNP	0.03		1.95		
TC19002396.hg.1	PNP	0.03	2.47			
TC06000526.hg.1	PNPLA1	0.01			1.51	
TC07003301.hg.1	PNPLA8	0.05				1.72
TC07001470.hg.1	POLR2J4	0.02			2.15	
TC05000624.hg.1	PPIF	0.04	2.71			
TC10000566.hg.1	PPIF	0.03		3.01		

Transcript Cluster ID	Gene Symbol	p-value	Fold expression			
			PMA		A23187	
			30 min	60 min	30 min	60 min
TC06000118.hg.1	PPP1R10	0.04	3.17			
TC06001502.hg.1	PPP1R10	0.04		2.06		
TC06002590.hg.1	PPP1R10	0.04	3.17			
TC09000165.hg.1	PPP1R10	0.04	3.17			
TC09000508.hg.1	PPP1R10	0.04	3.17			
TC12000975.hg.1	PPP1R10	0.04	3.16			
TC18000414.hg.1	PPP1R10	0.04	3.16			
TC19001224.hg.1	PPP1R10	0.04	3.26			
TC6_cox_hap2000137.hg.1	PPP1R10	0.04		2.02		
TC6_dbb_hap3000126.hg.1	PPP1R10	0.04		2.02		
TC6_mann_hap4000115.hg.1	PPP1R10	0.04		2.01		
TC6_mcf_hap5000116.hg.1	PPP1R10	0.04		2.01		
TC6_qbl_hap6000127.hg.1	PPP1R10	0.04		2.02		
TC6_ssto_hap7000118.hg.1	PPP1R10	0.04		2.02		
TC19000711.hg.1	PPP1R15A	0.01				2.40
TC01003732.hg.1	PPP1R15B	0.04				2.30
TC03000348.hg.1	PRKCD	0.04			2.63	
TC03002349.hg.1	PRKCD	0.04			3.85	
TC14001592.hg.1	PRMT5-AS1	0.04			1.84	
TC14001592.hg.1	PRMT5-AS1	0.05				2.13
TC04001625.hg.1	PRMT9; PRMT10	0.02		2.10		
TC20000051.hg.1	PRNP	0.02				1.53
TC01000641.hg.1	PRPF38A	0.05			1.87	
TC11000345.hg.1	PRR5L	0.04			2.32	
TC11002632.hg.1	PRR5L	0.05			1.54	
TC19000557.hg.1	PSMC4	0.04			1.92	
TC08002113.hg.1	PTCSC1	0.04			1.58	
TC10000625.hg.1	PTEN	0.05				1.84
TC14000306.hg.1	PTGER2	0.00				2.46
TC05000184.hg.1	PTGER4	0.02				2.70
TC01003638.hg.1	PTGS2	0.01			2.55	
TC10000535.hg.1	PTGS2	0.02	2.16			
TC01003006.hg.1	PTPN22	0.03		1.99		
TC10002355.hg.1	PTPRE	0.03			1.56	
TC07001347.hg.1	PURB; MIR4657	0.03				1.70
TC13000875.hg.1	RAB20	0.04			1.64	
TC09002922.hg.1	RALGDS	0.03		2.04		
TC03000762.hg.1	RASA2-IT1	0.05		1.65		1.77
TC04002922.hg.1	RASGEF1B	0.04			1.61	2.76

Transcript Cluster ID	Gene Symbol	p-value	Fold expression			
			PMA		A23187	
			30 min	60 min	30 min	60 min
TC01002289.hg.1	RCC2	0.03		1.62		
TC11003206.hg.1	RELA	0.04			2.33	
TC11003207.hg.1	RELA	0.04			1.50	
TC13000147.hg.1	RGCC	0.00				2.13
TC01001622.hg.1	RGS1	0.03		5.26		
TC17002011.hg.1	RGS1	0.01	2.69			
TC07000476.hg.1	RHBDD2	0.01				1.64
TC02000116.hg.1	RHOB	0.02			1.82	1.77
TC04000242.hg.1	RHOH	0.02				1.70
TC12000699.hg.1	RNA5SP365	0.01		1.66		
TC0X000797.hg.1	RNA5SP498	0.04			1.52	
TC0Y000114.hg.1	RNA5SP498	0.04			1.52	
TC04002415.hg.1	RNASE2	0.04	4.64			
TC19000647.hg.1	RNASE3	0.04	1.78			
TC08001120.hg.1	RNF122	0.03				1.59
TC08001600.hg.1	RNF139-AS1	0.00			2.05	
TC08002508.hg.1	RNF139-AS1	0.01			2.41	
TC01000388.hg.1	RNU11	0.01				2.15
TC22000358.hg.1	RNU12	0.00				2.10
TC14001226.hg.1	RNU2-14P	0.03		1.78		
TC02002066.hg.1	RNU2-63P	0.01				1.75
TC03000911.hg.1	RNU4-38P	0.04		1.56		
TC08000640.hg.1	RNU5E-3P	0.01	1.57			
TC01002206.hg.1	RNU5E-4P	0.05				1.84
TC08001642.hg.1	RNU6-1255P	0.04			2.12	
TC16001207.hg.1	RNU6-1262P	0.03			1.50	
TC04000679.hg.1	RNU6-531P	0.04			1.70	
TC10001616.hg.1	RNU6-780P	0.02	1.53			
TC03001814.hg.1	RNU6-789P	0.02			1.70	
TC12001085.hg.1	RNU7-103P	0.05			3.18	
TC06002665.hg.1	RNU7-124P	0.00	1.57			
TC07001484.hg.1	RNU7-189P	0.01	1.57			
TC01001142.hg.1	RNVU1-14	0.04				1.81
TC01003152.hg.1	RNVU1-15	0.04				1.75
TC01001136.hg.1	RNVU1-3	0.02			1.53	
TC01004733.hg.1	RP11-107M16.2	0.02			1.53	
TC03000853.hg.1	RP11-1149M10.2	0.01	5.92		1.59	
TC08000517.hg.1	RP11-1149M10.2	0.02		3.06	1.56	1.51
TC01001521.hg.1	RP11-160H22.3	0.00			2.71	2.70
TC19001402.hg.1	RP11-160H22.3	0.03	1.53			
TC17001702.hg.1	RP11-195E2.4	0.00	1.70			
TC07000803.hg.1	RP11-306G20.1	0.01		2.02		

Transcript Cluster ID	Gene Symbol	p-value	Fold expression			
			PMA		A23187	
			30 min	60 min	30 min	60 min
TC08000384.hg.1	RP11-318K15.2	0.04			6.73	
TC13000218.hg.1	RP11-327P2.5	0.03				1.86
TC08001169.hg.1	RP11-360L9.7	0.02			1.75	
TC08000531.hg.1	RP11-437J19.1	0.03	1.55			
TC01001048.hg.1	RP11-439A17.10	0.01			1.85	
TC01003987.hg.1	RP11-443B7.1	0.01			2.37	
TC01001943.hg.1	RP11-443B7.2	0.03			1.60	
TC01001676.hg.1	RP11-569A11.1	0.04			1.68	
TC04000898.hg.1	RP11-701P16.2	0.02			1.86	
TC01005851.hg.1	RP11-85G21.1	0.01			1.68	
TC19002172.hg.1	RP11-863K10.7	0.03	1.78			
TC22000317.hg.1	RP11-95H8.5	0.01	1.56			
TC02004282.hg.1	RP11-95K23.5	0.03	1.74			
TC07000385.hg.1	RP3-508I15.14	0.04	1.66			
TC22000306.hg.1	RP3-508I15.14	0.00		1.83		
TC22000305.hg.1	RP3-508I15.18; RP3-508I15.19	0.04		1.77		
TC0X001846.hg.1	RP4-562J12.2	0.04				1.63
TC01000207.hg.1	RP4-798A10.2	0.02			1.65	
TC02001763.hg.1	RPL9	0.04	1.57			
TC09002418.hg.1	RPS26	0.00	2.29			
TC15001878.hg.1	RPS26	0.01	3.89			
TC12000500.hg.1	RPS26; LOC100996747; LOC101929876	0.01		2.02		
TC10000988.hg.1	RPS26P11	0.04	1.57			
TC20000885.hg.1	RPS2P7	0.04				1.50
TC19000964.hg.1	RPS5	0.04				1.64
TC19000875.hg.1	RPS9	0.01			1.53	
TC19002231.hg.1	RPS9	0.03				1.60
TC09000089.hg.1	RRAGA	0.03				1.66
TC16001474.hg.1	RRN3P2	0.01		1.55		
TC02000034.hg.1	RSAD2	0.00		3.72		
TC02003008.hg.1	RSAD2	0.02	1.83	3.32		
TC21000946.hg.1	RUNX1-IT1	0.05			3.35	
TC19000541.hg.1	SAMD4B	0.02			1.67	
TC07001467.hg.1	SBDS	0.03				2.13
TC07000428.hg.1	SBDSP1	0.05				1.96
TC17000979.hg.1	SCARF1	0.03			1.52	
TC10000541.hg.1	SDAD1P1	0.01	2.17			
TC09000416.hg.1	SDF2	0.04	2.42			
TC09001733.hg.1	SEC16A	0.05				1.61
TC22000421.hg.1	SELO	0.04				1.54

Transcript Cluster ID	Gene Symbol	p-value	Fold expression			
			PMA		A23187	
			30 min	60 min	30 min	60 min
TC15001643.hg.1	SEMA7A	0.02		1.63		2.33
TC09002507.hg.1	SERF2	0.05	1.64			
TC20000867.hg.1	SERINC3	0.05			2.22	
TC06001220.hg.1	SERPINB9	0.02			5.56	
TC22000650.hg.1	SF3A1	0.04			2.09	
TC10001017.hg.1	SFMBT2	0.00		1.58		
TC06002126.hg.1	SGK1	0.02				1.84
TC03001380.hg.1	SHISA5	0.03				1.52
TC03001901.hg.1	SIAH2	0.00				1.61
TC21000506.hg.1	SIK1	0.02			1.54	
TC03000917.hg.1	SKIL	0.05		3.98		
TC02001300.hg.1	SLC11A1	0.03			1.87	
TC17001820.hg.1	SLC16A6	0.04			3.56	
TC17000780.hg.1	SLC16A6P1	0.04			3.32	
TC01000905.hg.1	SLC30A7	0.05		1.69		
TC12001426.hg.1	SLC38A1	0.04		2.05	1.61	
TC11000567.hg.1	SLC3A2	0.02		1.57		
TC11002708.hg.1	SLC3A2	0.03		2.44		
TC11003482.hg.1	SLC43A3	0.03		1.64		
TC04001810.hg.1	SLED1	0.03			4.75	2.94
TC04002881.hg.1	SLED1	0.03			6.06	3.31
TC04002881.hg.1	SLED1	0.00				
TC16001211.hg.1	SMPD3	0.04				1.76
TC20000395.hg.1	SNAI1	0.00			1.58	
TC13000632.hg.1	SNORA31	0.04			3.45	2.45
TC06000375.hg.1	SNORA38	0.04		1.58	1.54	
TC6_cox_hap2000071.hg.1	SNORA38	0.04		1.58	1.54	
TC6_mann_hap4000062.hg.1	SNORA38	0.04		1.58	1.54	
TC6_qbl_hap6000062.hg.1	SNORA38	0.04		1.58	1.54	
TC6_ssto_hap7000058.hg.1	SNORA38	0.04		1.58	1.54	
TC14000709.hg.1	SNORD114-29	0.03				1.50
TC06003807.hg.1	SNORD37	0.03	3.48			
TC17000230.hg.1	SNORD3A	0.01				4.79
TC17002061.hg.1	SNORD3A	0.04	2.06			
TC02004973.hg.1	SNORD3B-1	0.02	2.24			
TC16000855.hg.1	SNORD3B-1	0.02	2.24			
TC17000226.hg.1	SNORD3B-1	0.01				4.61
TC17001231.hg.1	SNORD3B-1; SNORD3B-2	0.01				4.61
TC17001238.hg.1	SNORD3C	0.01				4.40

Transcript Cluster ID	Gene Symbol	p-value	Fold expression			
			PMA		A23187	
			30 min	60 min	30 min	60 min
TC09000935.hg.1	SNORD3D	0.01	2.18			
TC17001233.hg.1	SNORD3D	0.05			1.71	
TC17001233.hg.1	SNORD3D	0.00		3.55		4.49
TC17000040.hg.1	SNORD61	0.04	1.51			
TC03000342.hg.1	SNORD69	0.05			1.82	
TC12000726.hg.1	SOCS2	0.04				1.52
TC17001917.hg.1	SOCS3	0.01			3.59	2.52
TC19000787.hg.1	SPACA6P	0.01			1.83	
TC01005360.hg.1	SPCS2P4	0.03		1.50		
TC01001721.hg.1	SRGAP2; SRGAP2D; SRGAP2B; SRGAP2C	0.04				1.54
TC01004687.hg.1	SRGAP2B	0.05				1.71
TC01000313.hg.1	SRRM1	0.04			1.79	
TC17002792.hg.1	SRSF2	0.03				1.75
TC17001908.hg.1	SRSF2; MIR636	0.02				1.73
TC05002109.hg.1	SRSF3	0.03	6.30			
TC06000530.hg.1	SRSF3	0.03		2.86		
TC06002757.hg.1	SRSF3	0.03		2.30		
TC15001906.hg.1	SRSF3	0.03	5.15			
TC02001757.hg.1	SRSF7	0.05				1.92
TC07000415.hg.1	STAG3L4	0.04			2.25	
TC07002389.hg.1	STAG3L4	0.03			2.14	
TC01005350.hg.1	STMN1	0.01				1.50
TC16000367.hg.1	STX4	0.01			1.57	1.58
TC17000166.hg.1	SUMO1P1	0.05	4.41			
TC20000959.hg.1	SUMO1P1	0.04		2.86		
TC06002270.hg.1	TAGAP	0.05				2.35
TC06003604.hg.1	TAP2	0.03			1.66	
TC06001279.hg.1	TBC1D7; LOC100130357; RP1-257A7.4	0.00		3.14		1.55
TC01002601.hg.1	TCTEX1D4; RP11-269F19.9	0.00				1.75
TC07000718.hg.1	TES	0.05		1.58		
TC13000426.hg.1	TFDP1	0.02		1.83	2.35	
TC13001318.hg.1	TFDP1	0.02		2.31	3.85	
TC12003253.hg.1	THAP2	0.03				1.60
TC04002943.hg.1	THAP9-AS1	0.04			1.91	
TC04002943.hg.1	THAP9-AS1	0.03				2.05
TC15000270.hg.1	THBS1	0.01		3.73	3.23	
TC22000728.hg.1	THBS1	0.00	3.99			
TC0X000238.hg.1	TIMP1	0.00			2.96	

Transcript Cluster ID	Gene Symbol	p-value	Fold expression			
			PMA		A23187	
			30 min	60 min	30 min	60 min
TC02001586.hg.1	TIPARP	0.02	2.50			
TC03000853.hg.1	TIPARP	0.00		2.20		
TC20000207.hg.1	TM9SF4	0.02			2.91	
TC14001318.hg.1	TMED10	0.03			1.80	
TC17001845.hg.1	TMEM55B	0.04	4.96			
TC17000118.hg.1	TMEM88	0.00		1.87	1.60	1.55
TC17001884.hg.1	TMEM88	0.01	2.20			
TC17000565.hg.1	TMUB2	0.03			1.67	
TC11003449.hg.1	TMX2	0.01			1.57	
TC06000371.hg.1	TNF	0.00				2.13
TC06002714.hg.1	TNF	0.00				2.45
TC6_apd_hap1000036.hg.1	TNF	0.01				2.40
TC6_cox_hap2000067.hg.1	TNF	0.00				2.13
TC6_dbb_hap3000058.hg.1	TNF	0.00				2.13
TC6_mann_hap4000059.hg.1	TNF	0.00				2.13
TC6_mcf_hap5000053.hg.1	TNF	0.00				2.13
TC6_qbl_hap6000058.hg.1	TNF	0.00				2.13
TC6_ssto_hap7000054.hg.1	TNF	0.00				2.13
TC05001946.hg.1	TNIP1	0.04			2.72	
TC05003310.hg.1	TNIP1	0.01			1.55	
TC09000237.hg.1	TPP1	0.05	2.49			
TC06001717.hg.1	TREM1	0.04			2.04	
TC06001732.hg.1	TRERF1	0.05				1.54
TC08000733.hg.1	TRIB1	0.03				2.43
TC03000814.hg.1	TSC22D2	0.04				1.81
TC21000341.hg.1	TTN	0.05	1.85			
TC21000501.hg.1	U2AF1	0.04				1.57
TC13001250.hg.1	UBAC2	0.04			2.33	
TC13000353.hg.1	UBAC2-IT1; RP11-461N23.5	0.04			2.04	
TC01004756.hg.1	UBAP2L	0.04			1.94	
TC12001625.hg.1	UBAP2L	0.04	3.30			
TC11002074.hg.1	UCP2	0.03				1.90
TC11001999.hg.1	UNC93B1	0.05				1.52
TC17002787.hg.1	UNK	0.00		2.22	2.66	1.89
TC22000149.hg.1	UPB1	0.05			2.04	
TC17001922.hg.1	USP36	0.00			1.53	
TC11002105.hg.1	UTS2	0.04	1.77			
TC06000608.hg.1	VEGFA	0.03		2.07		

Transcript Cluster ID	Gene Symbol	p-value	Fold expression			
			PMA		A23187	
			30 min	60 min	30 min	60 min
TC07001397.hg.1	VOPP1; LOC101060281	0.03			1.57	
TC0X000905.hg.1	VPS13A	0.04	1.51			
TC15000297.hg.1	VPS18	0.03		2.19		
TC17001984.hg.1	WDR45B; WDR45L	0.04			2.09	
TC17000309.hg.1	WSB1	0.05		2.51		
TC08001135.hg.1	XX-FW83128A1.2	0.02	2.46			
TC0X001529.hg.1	XX-FW83128A1.2	0.02		2.07		
TC02004620.hg.1	ZEB2	0.05		1.78		
TC14001153.hg.1	ZFP36	0.03	2.50			
TC19000544.hg.1	ZFP36	0.01		1.80	1.85	2.90
TC14001253.hg.1	ZFP36L1	0.04			2.15	
TC02001790.hg.1	ZFP36L2	0.04			2.31	
TC03002737.hg.1	ZNF107	0.05	2.54			
TC07000385.hg.1	ZNF107	0.00			2.74	1.81
TC0X001202.hg.1	ZNF107	0.02	4.13			
TC06000273.hg.1	ZNF165	0.01			2.66	2.11
TC08000866.hg.1	ZNF252P-AS1	0.05			1.51	
TC19000544.hg.1	ZNF638-IT1	0.04	1.50			
TC0X000219.hg.1	ZNF674-AS1	0.04			2.17	
TC19002391.hg.1	ZNF708	0.02			2.29	
TC19000390.hg.1	ZNF738	0.04			1.69	
TC06000274.hg.1	ZSCAN12P1	0.05		2.08		
TC06002667.hg.1	ZSCAN12P1	0.04		1.50		

Table S3. Number of connected transcripts regulated by specific transcription factors

Transcription factors	PMA -induced NOX -dependent NETosis		A23187 -induced NOX -independent NETosis	
	**30 min**	**60 min**	**30 min**	**60 min**
Androgen receptor	16		55	16
ATF-2/c-JUN		23		
C/EBP			52	
c-Myc	26	38	78	26
EGR1	9	18	49	9
ESR1 (nuclear)		24		
HIF1A		20	54	
IRF1			49	
NF-kB	10	14	57	16
P53	10	28		10
PPAR-b	9			13
PPAR-gamma		17		
PU.1			59	
RAR-a			66	
SP1			64	
STAT1		17		
STAT3	10	21	53	
STAT3				
STAT3				
STAT5			48	
TCF				10
TCF7L2 (TCF4)	10	16		
